# Association Between the Dietary Inflammatory Index (DII) and Head and Neck Cancer Incidence—A Narrative Review

**DOI:** 10.3390/nu18152421

**Published:** 2026-07-24

**Authors:** Starska-Kowarska Katarzyna

**Affiliations:** 1 Department of Physiology, Pathophysiology and Clinical Immunology, Department of Clinical Physiology, Medical University of Lodz, Żeligowskiego 7/9, 90-752 Lodz, Poland; katarzyna.starska@umed.lodz.pl; 2 Department of Otorhinolaryngology, EnelMed Center Expert, Drewnowska 58, 91-001 Lodz, Poland

**Keywords:** anti- and pro-inflammatory diet, dietary inflammatory index (DII), energy-adjusted dietary inflammatory index (E-DII), oesophageal cancer (ESCC), head and neck cancer (HNC), head and neck squamous cell carcinoma (HNSCC), laryngeal cancer (LSSC), nasopharyngeal cancer (NPSSC), oral cancer (OSSC), oropharyngeal cancer (OPSSC), upper aerodigestive tract cancers (UADT)

## Abstract

Head and neck cancer (HNC) comprises a heterogeneous group of tumours, most often of squamous cell origin, characterized by both high morbidity and mortality rates. It is the seventh most common form of cancer diagnosed in humans, accounting for approximately 4.7% of all cancers. Among HNCs, neck squamous cell carcinoma (HNSCC) is predicted to become the most common form of human cancer. Some sources include oesophagus (ESCC) in this group due to their similar histology, being described as upper aerodigestive tract cancers (UADT). Unfortunately, 60–70% of cases are diagnosed late, i.e., at clinical stages III-IV. As a result, despite modern surgical techniques and oncological treatments, the survival rate remains below 40–60% due to frequent lymph node metastases and local tumour recurrence. There is a growing concern that diet and inflammatory dietary components may influence the initiation and development of HNSCC. The inflammatory potential of diets can be quantified by the Dietary Inflammatory Index (DII). The DII was derived from an analysis of 45 dietary constituents that either increase or decrease inflammation. Several recent clinical studies have noted a significant relationship between DII score and many inflammation-associated chronic diseases, such as obesity, cardiovascular and neurodegenerative disorders, and diabetes, and the incidence of various human cancers, i.e., prostate, ovarian, breast, colorectal cancer, and HNC. However, few studies have investigated the relationship between DII and HNSCC, with most being limited to observational, case-control, and cross-sectional studies. Therefore, the aim of this narrative review is to present the substantial oncological aspects of DII, discuss the use of DII and its modification, the Energy-Adjusted Dietary Inflammatory Index (E-DII), as indicators of HNSCC risk. It also introduces key diet-induced pro- and anti-inflammatory mechanisms and the cellular molecular signalling pathways determining the carcinogenesis of HNSCC. It provides a comprehensive overview of the current literature, including key opinion-forming systematic reviews, as well as molecular, observational, cross-sectional and case-control studies, all of which are accessible via scholarly databases such as PubMed/EMBASE/Web of Science. Thus, the work serves as a compendium of up-to-date knowledge on the relationship between DII/ED-II score and HNSCC etiopathogenesis and the influence of a diet-induced persistent inflammatory microenvironment.

## 1. Introduction

Head and neck cancers (HNC) comprise those that begin on the mucosal surfaces of the head and neck region, including the oral cavity, nasopharynx, oropharynx, hypopharynx, larynx, salivary glands, nasal cavity and paranasal sinuses. The most common histological type of HNC is head and neck squamous cell carcinoma (HNSCC), originating in the squamous cells that line the mucosa of the head and neck organs [1,2]. Some sources classify oesophageal cancer (ESCC) as head and neck cancer (HNC) based on its similar histology: the tumour is also a squamous cell carcinoma arising from the squamous epithelium of the upper gastrointestinal tract, i.e., the cervical part of the oesophagus, which is histologically identical to the epithelium lining the upper respiratory tract. As these cancers derive from anatomically related organs with shared risk factors, *viz.* smoking and regular alcohol consumption, head and neck cancers are also referred to as upper aerodigestive tract cancers (UADT) [3].

A recent multi-year, multi-population study from the Global Cancer Statistics Group (GLOBOCAN 2022) found HNSCC to constitute the seventh most common cancer globally, accounting for ~4.7% of human malignancies, the condition is newly diagnosed in over 946,456 patients and contributes to nearly 482,001 deaths [4]. The tumour mainly affects men, who are at two to fourfold higher risk than women for developing HNSCC. It has been predicted that the incidence of HNSCC will increase by 30% by 2030, resulting in an estimated 1.08 million new cancer cases per year [5,6,7]. Oesophageal cancer is also widespread: it is the eleventh most commonly diagnosed cancer and the seventh cause of death worldwide; it constitutes 2.6% of all cancers globally, and is responsible for 510,716 new cases and 445,000 deaths. Also, the cancer is diagnosed two to three times more often in men than in women, and its highest incidence rate is observed in Eastern Asia and Eastern Africa.

Histologically, squamous cell carcinoma represents one-third of oesophageal cancer subtypes. HNSCC and ESCC together constitute ~7% of all human cancers: approximately 1.3 million UADT cancer cases and 830,000 deaths were reported in 2022 [4,8]. Unfortunately, despite the use of modern surgical procedures and advanced combined oncological treatment methods, the five-year survival rate of patients with HNSCC remains below 40–60%. This is mainly due to delayed diagnosis, with most cases detected at WHO stages III and IV. The diseases are also characterised by rapid development of neoplastic lesions and aggressive course, as well as frequent lymph node metastases, local tumour recurrence and chemoradioresistance [8,9].

According to the National Comprehensive Cancer Network (NCCN), the best-known risk factors for the development of HNSCC include tobacco smoking, alcohol consumption, exposure to environmental pollutants; occurrence is also associated with chewing areca nut products, including ‘betel quid’ and betel leaf, as well as slaked lime, and poor oral hygiene [10,11]. The interaction of these common risk factors can increase the risk of HNSCC diagnosis nearly 35-fold. Cigarette smoke contains approximately 7000 chemicals, including 70 known carcinogens such as benzo[a]pyrene, a key polycyclic aromatic hydrocarbon (PAH), tobacco-specific nitrosamines (TNA), and N′-nitrosonornicotine (NNN) [12,13,14].

According to the Cancer Genome Atlas Network (TCGA), tobacco-related cancers are characterized by aberrations in key regulatory genes: those regulating the cell cycle (*CDKN2A* and *CCND1*), those determining cell proliferation and survival (*TP53*, *HRAS*, *PIK3CA*, and *EGFR*), those controlling cell differentiation (*NOTCH1*), and a gene regulating the WNT signalling pathway [5]. During the initiation and development of neoplastic lesions, they may exhibit deletion of chromosome 9p, responsible for the inhibition of p16^INK4a^ protein (CDKN2A) expression, and replication of chromosome 7p, leading to overexpression of the epidermal growth factor receptor (EGFR) [15,16]. Equally frequently described are mutations leading to the activation of proto-oncogenes, i.e., *c-myc*, *RAS* family genes, *ERB-B*, *BRAF*, *HER-2*, *c-KIT*, *BCL-2*, *STAT3*, and aberrations inhibiting the activity of tumour suppressor genes, anti-oncogenes, i.e., *RB1*, *TP53*, *PTEN*, *CDKN2A*, *INK4* [5,17].

Similarly, alcohol and tobacco use are believed to play key roles in more than half of new ESCC cases. Both ethanol and its metabolite acetaldehyde primarily induce ESCC through DNA damage and inflammation [18,19,20]. It is important to note that ESCC has been found to be more common in Asian populations, who are more likely to be characterised by the slow *ALDH2* variant (rs671) (30–50% of East Asian patients) than European populations (5%). This variant inhibits the conversion of acetaldehyde to acetic acid, leading to prolonged epithelial exposure to the ethanol metabolite [21,22].

In recent years, attention has also been drawn to the growing incidence of squamous cell carcinoma (SCC) of the throat and larynx following infection by oncogenic strains of human papillomavirus (HPV), mainly HPV16/18. Importantly, this type of oropharyngeal cancer (OPSCC), which accounts for as many as 38–80% of new malignant HNSCC lesions, affects young individuals with a good economic status [23,24]. HPV16/18 E6 and E7 viral antigens are oncoproteins/oncogenic factors that promote the initiation and further development of the tumour. The E6/E7 proteins inactivate two tumour suppressor proteins, p53 and pRB1, respectively, ultimately resulting in unregulated cell division, cell growth, and cell survival [25,26,27]. E6 also leads to deregulation of the *c-myc* oncogene; this induces transcription of the catalytic subunit of human telomerase (hTERT), which disrupts the function of CDKs, cyclins, and E2F transcription factors. Also, Myc has been found to reverse the inhibitory effects of CDK inhibitors such as p27^KIP1^ and p21^CIP1/WAF1^ [17,26]. According to TCGA, typical gene mutations for HPV-associated cancers include *PIK3CA*, *FGFR*, *DDX3X* and *CYLD* [28]. HPV-induced tumours also exhibit amplification in chromosome 3q and the loss of chromosomes 11q, 13q, 14q, 16p and 16q [15,16].

It is well known that the diet has a strong influence on the inflammatory response in the human body. Furthermore, recent studies have noted that specific dietary components can encourage or inhibit the persistent inflammatory microenvironment responsible for the development of certain chronic lifestyle diseases. As a result, various tools have been developed to assess the relationship between the presence of chronic, “simmering” body inflammation and lifestyle disorders. However, numerous studies have focused on individual dietary components, ingredients, and nutrients; this has limited their potential to study the real synergistic effects of multiple dietary factors. In response, new studies are applying various scales and dietary patterns, like Dietary Inflammatory Index (DII), Healthy Eating Index-2015 (HEI-2005), Alternative Healthy Eating Index (AHEI-2010), Mediterranean Dietary Score (MDS), HNC-specific MDS (MDS-HNC), Mediterranean Dietary Pattern Adherence Index (MDP), Mediterranean Adequacy Index (MAI), alternate Mediterranean Diet Score (aMED). These tools consider the interaction of nutritional products and dietary components, which may have a significant impact on the development of chronic conditions that may induce persistent inflammation [29,30,31,32,33,34,35].

Among these tools, the Dietary Inflammatory Index (DII), developed in 2009, deserves particular attention due to its widespread use, global popularity, and scientific recognition [36]. The DII is an evidence-based, numerical tool designed to quantify the inflammatory potential of various individual diets and food ingredients developed based on dietary intake data. It was derived from an analysis of the inflammatory effect of 45 dietary components and food parameters from 11 countries, including macronutrients, micronutrients, and bioactive compounds. Higher positive DII scores specify high pro-inflammatory diets/foods, while lower DII results designate high anti-inflammatory diets/nutrients [37]. A considerable body of clinical research has confirmed a significant relationship between DII category and many inflammation-associated diseases, such as obesity, metabolic syndrome, cardiovascular disorders, neurodegenerative diseases and diabetes [38,39,40,41,42,43]. Importantly, numerous observational studies and meta-analyses also indicate that diet-associated inflammation and a high DII category are associated with increased risk of many human cancers, including prostate, ovarian, breast, colorectal, gastric, oesophageal, pancreatic, urologic cancer and HNC [44,45,46,47,48,49,50,51,52,53,54]. However, the precise relationship between DII and human carcinogenesis, including HNC or UADT, appears inconsistent [40,55,56,57]. Moreover, to ensure the accuracy of nutritional assessment and increase its practical value, the study uses food frequency questionnaires (FFQs) with varying numbers of dietary products, together with advanced research tools for assessing habitual eating habits at a given time [58].

This narrative review presents the substantial oncological aspects of the DII and its modification (the energy-adjusted dietary inflammatory index; E-DII) and discusses their use as indicators of the risk of HNSCC and ESCC. It also introduces the key pro- and anti-inflammatory mechanisms associated with diet, and the cellular molecular signalling pathways determining upper aerodigestive tract (UADT) carcinogenesis. It includes articles concerning diet and HNSCC risk published before 2024; importantly, the last published meta-analysis (Shrivastava et al. 2024) covers the period until 2020 [59]. A number of other articles written after 2020 were identified using scholarly databases such as PubMed/EMBASE/Web of Science. Therefore, this work is a valuable compendium of knowledge on the etiopathogenesis of HNSCC related to the DII/E-DII scores and diet-induced inflammation; it also serves as a summary of all studies and knowledge regarding HNSCC risk estimation based on DII/E-DII indicators up to 31 May 2026.

### 1.1. Dietary Inflammatory Index (DII) and Energy-Adjusted Dietary Inflammatory Index (E-DII)

The growing awareness of the relationship between diet and chronic disease associated with persistent inflammation has resulted in the design of various dietary indicators and indexes for clinical practice. For many years, diet specialists have asserted that a pro-inflammatory diet can contribute to and activate an intense chronic inflammatory response, resulting in an increased risk of many lifestyle diseases, such as obesity, cardiovascular disorders and cancers [38,39,40,41,42,43].

Initial data on the potential of dietary components and nutritional products to modulate a persistent inflammatory microenvironment were first obtained from a longitudinal observational study at the University of South Carolina’s Arnold School of Public Health [60]. The study consisted of a review of 927 articles examining the relationship between various food products and serum high-sensitivity (*hs*) C-reactive protein (CRP) levels, an inflammatory marker, in 600 adults. The results indicate that the consumption of anti-inflammatory diets and dietary nutrients was related to a decrease in CRP level. Conversely, diet-induced inflammation led to a significant increase in serum *hs*-CRP protein values. This study resulted in the creation and validation of the Inflammatory Index Score (INS).

#### 1.1.1. Dietary Inflammatory Index (DII)

Subsequent years saw more accurate assessment of the relationship between diet and inflammation and overall health. The development of the DII required extensive searches of nearly 6500 articles published between 2008 and 2010, of which 1943 were ultimately selected. The DII database and the scoring system, whether in the form of categorical cut-points (such as tertiles and quartiles) or based on a continuous DII score, were based on the inflammatory effects of various foods and individual nutrients. Finally, in 2014, after an analysis of articles including both cell cultures and observational and epidemiological studies, Shivappa et al. [37] introduced a new literature-derived, population-based algorithm for calculating the DII. This new method was based on the relationship of individual nutritional products and food parameters with six key markers of inflammation, i.e., serum levels of pro-inflammatory (IL-1β, IL-6, TNF-α and CRP protein) and anti-inflammatory substances (IL-4 and IL-10).

The newly-developed DII standardized individual intakes based on the global mean (and standard deviation). It incorporated 11 different data sets reflecting real-world human consumption in different countries (USA, Australia, the Kingdom of Bahrain, Denmark, India, Japan, New Zealand, Taiwan, South Korea, Mexico, and the United Kingdom) and the inflammatory potential of their diet.

Briefly, the index used standardized intake scores, i.e., Z scores, which were converted to proportions. These were multiplied by their inflammatory effect scores and then summed. This yielded an overall DII score for an individual diet. Each food product was rated on a scale of (−1), i.e., anti-inflammatory, to (+1), i.e., pro-inflammatory; a score of (0) indicates that the food has no net effect on inflammation. The final score was weighted based on the level of scientific evidence. Therefore, the inflammatory potential of specific nutrients, foods, and diets indicates their ability to induce and sustain, or inhibit inflammatory reactions in the body.

The DII enables the evaluation of diet based on the consumption of 45 distinct pro- and anti-inflammatory food types. Its score ranges from +7.98, indicating a maximally pro-inflammatory diet, down to −8.87, indicating a maximally anti-inflammatory diet [37]. In its current form, the DII is an important nutritional tool used to analyse the inflammatory potential of the entire diet, not just individual nutrients, based on their likely contribution to regulating inflammation in the body; thanks to its global nature, it is suitable for testing a range of individuals and populations, regardless of region, population type, or ethnicity. Interestingly, although standard analyses of DII include 45 dietary components, some only employ the 28 available in the National Health and Nutrition Examination Survey (NHANES) database. Despite this, the DII index based on these 28 components is a well-documented analysis tool that also accurately reflects diet-related inflammation [61,62].

##### Index DII Calculation Procedure

- Collecting Intake Data: The precise daily intake of individual nutrients is determined using a daily food intake diary or a food frequency questionnaire (FFQ).
- Comparison with a Global Database (Z-Score): The individual intake of each nutrient is analysed in relation to a standard global database representing the mean intake and standard deviation in the population of 11 countries. The Z-Score is calculated using the following formula:
  $$Z=\frac{Individual\;Intake-Global\;Mean}{Standard\;Deviation}$$
- To minimize errors resulting from distribution asymmetry (right skewing), the Z-value is converted to a cumulative probability (percentile), which is then used to calculate a centered percentile value thus:
  Centered percentile = (Percentile Score × 2) − 1
  This results in a symmetrical distribution ranging from −1 to +1.
- Multiply by the Inflammatory Effect Score: The resulting percentile is multiplied by the inflammatory weight assigned to the ingredient (overall food parameter—specific inflammatory effect score). This weight reflects the impact of the component on six inflammatory markers (IL-1β, IL-4, IL-6, IL-10, TNF-α, and CRP).
  Food Parameter DII Score = Centered Percentile × Inflammatory Effect Score
- Sum All Component Scores: The DII for a given individual is the sum of the scores for all available nutrients.
  Overall DII Score = Σ Food Parameter DII Scores

A previous study by Cavicchia et al. [60] evaluated the practical application of the DII test in conjunction with the assessment of high-sensitivity CRP (*hs*-CRP) levels, using data from the Seasonal Variation of Blood Cholesterol Study (SEASONS) longitudinal observational study. Interestingly, while no significant correlation was observed with *hs*-CRP levels used as a continuous variable, a significant and inverse correlation was noted when *hs*-CRP was used as a dichotomous dependent variable (≤3 mg/L, >3 mg/L). This result indicates a nonlinear relationship between *hs*-CRP and diet.

The inflammatory potential of a diet has been found to vary with human population and regional dietary patterns, and has been attributed to the consumption of specific nutrients, foods and dietary constituents. The components are believed to regulate each other’s pro- or anti-inflammatory properties, thus promoting or suppressing any chronic inflammatory status in the body. Importantly, the search for healthy food ingredients has gained significant interest among researchers, and the last few years have seen a significant increase in the number of publications on the topic. These studies highlight the protective properties of anti-inflammatory nutrients and the benefits of eating a healthy diet, i.e., one based on unprocessed or minimally preserved or modified foods rich in antioxidants, anthocyanidins, flavonoids, polyphenols, carotenoids, polyunsaturated fatty acids (particularly marine ω-3 PUFAs), fibre and natural spices, as well as vitamins and some trace elements. Such diets include certain key components, *viz.* oily fish (salmon, mackerel, sardines, herring); leafy greens and other vegetables (spinach, kale, arugula, broccoli, tomatoes, red cabbage, red onion); healthy fats (extra virgin olive oil, avocado, flaxseed oil); nuts and seeds (walnuts, almonds, chia seeds, flaxseeds); whole grain products (whole-wheat rye bread, oatmeal, brown rice, groats); green tea, cocoa/dark chocolate; legumes (lentils, chickpeas, soybeans); fermented dairy products (kefir, yogurt) and berries (blueberries, raspberries, strawberries, blackberries, and currants). A number of studies have focused on the hypothesis that a lower DII score indicates an anti-inflammatory diet, and would hence be associated with a reduced incidence of cancer in humans [36,63,64,65,66,67,68]. Some well-known anti-inflammatory diets include the Mediterranean diet (MD), macrobiotic diet and plant-based (PB) diet [68].

In contrast, a number of foods and dietary constituents have known pro-inflammatory properties. These include oxidized lipids, saturated fatty acids (SFAs) commonly found in red and processed meat and full-fat dairy products, trans fatty acids found in partially hydrogenated oils, refined carbohydrates found in sugary snacks, refined cereals, sodas/sodium, food additives and emulsifiers, e.g., carrageenan. Importantly, numerous studies indicate a significant association between more pro-inflammatory diets, i.e., with higher DII results, and increased risk of human cancers [44,45,46,47,48,49,50,51,52,53,54]. In addition, numerous epidemiological and observational studies report increasing consumption of foods labelled as processed or ultra-processed (UPFs) by the NOVA food classification system. Their consumption is also believed to support inflammatory processes, have a negative impact on the gut microbiota and increase the risk of diet-related lifestyle diseases [69]. Typical examples of pro-inflammatory diets are the Western diet (WD), high-fat diet (HFD) and the fast-food diet. Importantly, these processed foods are high in energy and have been treated with flavourings, chemicals and salt to improve the taste. They are typically characterized by higher intakes of pro-inflammatory food products, such as refined grains and simple sugars, and higher levels of pro-inflammatory advanced glycation end-products (AGEs) that increase glycaemic load and disrupt gut microbiota composition. Pro-inflammatory diets and UPFs may also include considerable amounts of additives and emulsifiers (e.g., carrageenan) implicated in the inflammatory cascade. They often contain contaminants from plastic food packaging, such as bisphenol and phthalates, which are also known to be endocrine disruptors and potential carcinogens [40,70,71,72,73,74,75,76].

The oral microbiota can influence the development of head and neck cancers due to imbalances within the microbiota of the oral cavity and gut (including *Streptococcus mitis*, *Haemophilus parainfluenzae*, and *Actinobacteria phylum*) and pathogens (including *Fusobacterium nucleatum*, *Porphyromonas gingivalis*, *Streptococcus anginosus*, *Treponema denticola*, *Prevotella intermedia*, *Peptostreptococcus stomatis*, and *Parvimonas micra*). This dysbiosis leads to the activation of chronic inflammation, inhibits apoptosis, and contributes to the production of toxic and carcinogenic metabolites. The oral microbiota is the second most diverse microbial ecosystem in the human body, encompassing over 700 species of bacteria, together with fungi and viruses. The risk factors typical for UADT, such as smoking, alcohol abuse and poor hygiene, can encourage the proliferation of pathogenic flora [77,78]. Indeed, considerably higher numbers of pathogenic bacteria have been identified in the saliva of HNSCC patients and in tumour tissues [79,80]. In HNCs, carcinogenic microbiota is believed to act by inducing chronic inflammation, supporting the formation of carcinogenic metabolites, and suppressing the immune system. Bacteria such as *F. nucleatum* and *P. gingivalis*, identified in both the oral cavity and the intestinal microflora, stimulate toll-like receptors such as TLR4, resulting in the activation of the key inflammatory transcription factor NF-κβ. Its induction leads to the continuous production and secretion of pro-inflammatory cytokines such as IL-6, IL-8, TNF-α and matrix metalloproteinases (MMPs); it also increases the production of ROS/RNS, resulting in oxidative stress, and causes DNA damage [81,82,83,84,85,86].

The adhesin FadA, associated with *F. nucleatum* colonization of mucosa, activates E-cadherin/β-catenin signalling pathways, directly stimulating cancer cell proliferation and migration. *P. gingivalis*, the cause of smouldering periodontitis, may inhibit epithelial cell apoptosis by activating the JAK1/STAT3 pathway and induce epithelial-mesenchymal transition (EMT), which promotes metastasis and generates resistance to chemotherapy in HNSCC [81,82,83,84,85,86]. *S. anginosus* also increases the conversion of ethanol to acetaldehyde, a potent carcinogen, damages DNA structure, and stimulates VEGF-mediated angiogenesis [81,82,83,84,85,86]. Salivary acetaldehyde levels are also drastically increased by smoking, which may have a synergistic effect with alcohol on tumour formation. Other bacteria, such as *P. aeruginosa*, can convert nitrates into nitric oxide (NO); at high concentrations, this modulates metastasis and blocks the cell cycle [81,82,83,84,85,86].

Furthermore, excessive growth of harmful bacteria damages the epithelium, leading to what is known as “leaky gut.” In such cases, toxins and bacterial particles (e.g., LPS) penetrate the bloodstream, triggering a persistent, systemic inflammatory response. Finally, dysbiosis also modifies the tumour microenvironment and immunocompetent cells toward immunosuppression. Oral pathogens degrade immunoglobulins and complement components using proteolytic enzymes, such as gingipain in *P. gingivalis*, allowing tumour cells to escape from immune surveillance.

Various modified versions of the DII have been developed, including the Dietary Inflammatory Score (DIS), Inflammatory Dietary Pattern (IDP), Empirical Dietary Inflammatory Patterns (EDIP), Lifestyle Inflammatory Score (LIS), Anti-Inflammatory Dietary Index (AIDI) and Food-based Index of Dietary Inflammatory Potential (FBDI). The most frequently used version is the energy-adjusted Dietary Inflammation Index (E-DII), a valuable, validated tool in nutritional epidemiology and clinical practice [87,88,89,90,91,92]. Importantly, the studies on these “adapted DII” tools demonstrate considerable heterogeneity, with some omitting certain pro-inflammatory parameters, and others analysing varying numbers of nutritional factors, calculating z-scores differently, and using means and standard deviations from a global database of population studies. Furthermore, many studies omitted other important lifestyle factors (e.g., physical activity, smoking, alcohol consumption) and body mass index (BMI). However, the introduction of a well-validated tool, the food frequency questionnaire (FFQ), and other lifestyle questionnaires in 2019 allowed for the selection of food groups thought to have an impact on persistent inflammation and included bioactive substances [36,87,88,89,90,91,92,93,94].

#### 1.1.2. Energy-Adjusted Dietary Inflammatory Index (E-DII)

It should be noted that the most useful indicators, DII and E-DII, also differ significantly in terms of methodology, the way they consider energy intake, and their practical application. The DII primarily focuses on the overall inflammatory potential of dietary patterns without considering total energy intake, which can bias results in populations with varying energy intake levels, while the E-DII includes energy intake; as such, it can provide a more accurate insight into the contribution of dietary components to inflammation relative to total caloric intake [36]. The E-DII was developed using an energy density approach, which calculates nutrient-adjusted and final energy-adjusted DII scores per 1000 calories consumed. Hence, the tool can account for the relationships between energy and nutrient intake in both healthy individuals and those with pathophysiological processes [90].

The E-DII index is therefore an advanced scientific tool used in epidemiology and dietetics to quantitatively assess the inflammatory potential of a person’s entire diet. By taking into account the intake of individual nutrients per 1000 kcal, the E-DII eliminates the error resulting from the fact that people who eat larger amounts of food automatically consume more of both pro- and anti-inflammatory compounds [36,90,95]. In practice, algorithms are used to analyse dozens of different dietary parameters for their impact on inflammatory markers.

Importantly, the E-DII is a very practical scientific tool for quantitatively assessing the inflammatory potential of a person’s diet in relation to calorie intake. It is used as a potential comparable indicator in epidemiological studies that require adjustment for energy intake, including studies involving obese individuals, or for comparing the results of multicentre studies. A high E-DII score (a highly pro-inflammatory diet) is strongly linked in scientific research to an increased risk of developing many chronic conditions, such as cardiovascular disease and hypertension, type 2 diabetes and metabolic syndrome, certain types of cancer, and obesity. Furthermore, estimating E-DII allows for modifications to the daily diets of healthy individuals and those with conditions such as obesity to effectively lower E-DII and support the body in combating underlying inflammation [36,61,62,95,96].

Hence, the E-DII index has demonstrated clinical significance in chronic disorders such as cardiovascular disease, diabetes, and cancer [61,62]. Importantly, by taking energy levels into account, E-DII data can contribute clinically to dietary modifications that may reduce disease risk [96]. Furthermore, this approach eliminates the problem of interpreting results, reducing the effects of individual differences in energy intake on the findings. Thus, E-DII provides a more accurate picture of the influence of diet on inflammatory potential, and plays an important role in introducing dietary interventions into clinical practice that could reduce the risk of chronic diseases [36]. However, the specific relationship between the E-DII index and carcinogenesis remains largely unexplored.

While observational, case-control, and cross-sectional population-based studies have yielded varying findings, the DII and/or E-DII nevertheless play parts in identifying appropriate dietary goals for individuals that can reduce the risk of many chronic conditions, and in establishing global eating patterns and promoting a healthy lifestyle [38,39,40,41,42].

**Figure 1:**
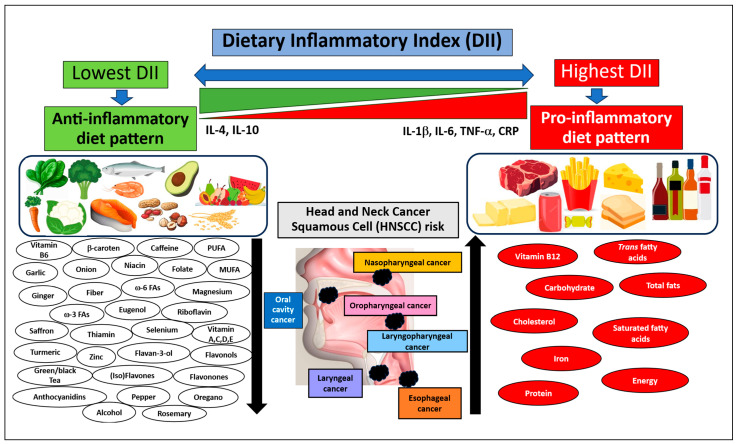
Dietary ingredients, nutritional products and food parameters included in the Dietary Inflammatory Index (DII). The DII was developed based on blood concentrations of pro-inflammatory biomarkers (IL-1β, IL-6, TNF-α, CRP) and anti-inflammatory cytokines (IL-4 and IL-10). DII scores of foods and dietary constituents are described based on their pro- or anti-inflammatory potential. The DII assesses up to forty-five food parameters, including macronutrients, vitamins, and minerals, ranging from highly pro-inflammatory (positive scores) to highly anti-inflammatory (negative scores). A negative DII result indicates an anti-inflammatory diet rich in antioxidants and fibre. The positive DII score indicates an inflammatory diet rich in saturated fats found in red meat, full-fat dairy products, refined carbohydrates and processed foods. The relationship between DII score and the head and neck cancer (HNC) incidence is described. A high DII score highlights a higher risk of HNC, while a low DII score indicates a reduced risk. Abbreviations: MUFA: monounsaturated fatty acid; PUFA: polyunsaturated fatty acid; ω-3 FAs: omega-3 fatty acids; ω-6 FAs: omega-6 fatty acids. The figure constitutes the exclusive intellectual property of the author of the work. Primary sources used to create the Figure: [36,61,62,95,96].

The pro- and anti-inflammatory dietary components and nutritional products included in the dietary inflammatory index (DII) are shown in Figure 1.

### 1.2. Basic Aspects of Pro- and Anti-Inflammatory Diets and Their Potential Relationship with Carcinogenesis

#### 1.2.1. Pro-Inflammatory Diet

Recent research indicates that unhealthy dietary habits and the use of pro-inflammatory diets, such as the Western Diet (WD), High-Fat Diet (HFD), and fast-food diet, may contribute to the development of lifestyle diseases, including HNSCC cancer [53,97,98,99]. These diets are characterized by high consumption of highly processed foods, which can be rich in pro-inflammatory food products. Such products include refined grains, simple and refined sugars, conventionally raised animal products, such as red and processed meat, eggs and high-fat dairy, high-sugar drinks, sweets and fried foods. They are also rich in food additives, like emulsifiers and sweeteners, and compounds generated by production or heat treatments during processing; these can include nitrogenated compounds (nitrates and nitrites), heterocyclic amines and polycyclic hydrocarbons, oxidized lipids, lipopolysaccharides (LPS) and iron, which increase the risk of overall low-grade chronic inflammation (i.e., metaflammation or diet-induced inflammation) and potential carcinogenesis.

Unfortunately, such diets are also often characterised by excess energy in relation to need, particularly in industrialized countries. Many studies also indicate that such diets are pro-inflammatory, which has a significant impact on host-immune reactions, metabolism, mitochondrial function and oxidant status in the human body, and on the gut microflora. The presence of inflammatory factors in serum, such as CRP, IL-1β, IL-6, and TNF-α, resulting from the use of pro-inflammatory nutrition, has been associated with high DII values, allowing the identification of foods that promote inflammation in the daily diet of various populations around the world [53,100,101,102].

The regular consumption of high-fat and high-carbohydrate diets also leads to weight gain and obesity. Epidemiological studies also indicate that high BMI, a common indicator of physical health, can favour carcinogenesis by driving epigenetic modifications such as DNA methylation and histone modification; these may influence proto-oncogene activity and silence tumour suppressor genes [103]. In the last decade, in an effort to curb the increase of lifestyle diseases, the World Health Organization (WHO) has increasingly recommended avoiding diets that promote and sustain persistent inflammation (https://www.who.int/news-room/fact-sheets/detail/obesity-and-overweight; accessed on 31 May 2026) [104].

Importantly, the daily consumption of unhealthy pro-inflammatory nutrition is directly associated with the accumulation of adipose tissue and body fat. The resulting increase in immunologically highly active adipocytes is associated with a high risk of comorbidities such as cardiometabolic diseases and cancers. The resulting obesity can also cause adipose tissue dysfunction by promoting immune cell infiltration, impaired angiogenesis, local hypoxia, fibrosis, and unregulated production of cytokines and adipokines, as well as increased oxidative damage and disrupted gene transcription. In contrast, patients with cancer, particularly in the head and neck region, frequently report consuming a limited and nutritionally-poor diet with low levels of fruits, vegetables, and vitamins. Such diets can interfere with immunocompetent cell activity and alter important metabolic pathways due to deficiencies in anti-inflammatory nutrients [105,106,107,108,109,110,111,112].

Food components such as total fatty acids, saturated fatty acids (MUFA, PUFA), *trans* fatty acids and cholesterol can activate adipose tissue cells (adipocytes) and adipose tissue macrophages and enhance oxidative stress factor production. Interestingly, the resulting death of adipocytes due to tissue hypoxia can exacerbate inflammation and further worsen metabolism. Moreover, dead adipocytes release free lipid droplets of cholesterol and cytotoxic fatty acids, which are key indicators of inflammation in adipose tissue; these create crown-like structures (CLS) and need to be eliminated by macrophages. Interestingly, during chronic inflammation, rather than removing dead cells, many macrophages accumulate around necrotic clusters. This leads to the release of pro-inflammatory cytokines (e.g., TNF-α, IL-1β) and the further development of a chronic, low-grade inflammatory microenvironment. These are accompanied by the release of alarm signals, i.e., damage-associated molecular patterns (DAMPs) and free fatty acids, activating the cells of the immune system [113,114,115,116,117].

The adipocytes and macrophages induced by pro-inflammatory diet components such as linoleic acid and saturated fats like stearic acid effectively enhance the infiltration of pro-inflammatory immune cells, such as Th1 CD4^+^ T cells, CD8^+^ cytotoxic T lymphocytes (CTLs), Th17 T cells, monocytes, natural killer cells/invariant natural killer cells (NK/iNKT), and mast cells. They also inhibit the proliferation of anti-inflammatory cells, i.e., Th2 CD4^+^ T cells and regulatory T lymphocytes (CD4^+^CD25^+^Foxp3^+^Tregs), leading to decreased production of anti-inflammatory cytokines such as IL-4, IL-10 and transforming growth factor-beta (TGF-β). These phenomena are also intensified by the secretion of adipokines and leptin by adipocytes [109,111,118,119,120].

Under the influence of free radicals, nitric oxide (NO), and reactive oxygen species and reactive nitrogen species (ROS/RNS), adipocytes support macrophage attraction and stimulation, and their shift towards the pro-inflammatory M1 subpopulation or classically-activated macrophages. This is made possible by molecules such as monocyte chemoattractant protein-1 (MCP-1, CCL2) and regulated on activation, normal T cell expressed and secreted (RANTES, CCL5) [115,116,121,122]. Neutrophils also contribute to macrophage and adipocyte activation through the secretion of cathelicidins and IL-1β, which attract monocytes and promote their differentiation into M1-type macrophages. They also enhance neutrophil extracellular traps (NETs), which directly stimulate macrophages to polarize towards M1, and activate the NLRP3 inflammasome, which intensifies the inflammatory cascade and promotes the release of inter alia elastase, myeloperoxidase (MPO), and IL-1β. Activation of the M1 macrophage subpopulation, through increased production of IFN-γ, TNF-α, IL-1β, IL-6, IL-12, and monocyte chemotactic protein 1 (MCP-1), increases the proliferation and formation of pro-inflammatory immunocompetent cells. This also results in the increased proliferation and production of Ig antibodies by B cells [120,123,124,125,126].

The consumption of a diet rich in fats and carbohydrates leads to a reduced number of iNKT cells. They stop functioning as “guardians” of adipose tissue, and their loss correlates with the influx of pro-inflammatory macrophages, which maintain a state of chronic inflammation [127,128,129]. Th1 lymphocytes, a specialized subset of CD4^+^ T lymphocytes that lead the cellular immune response, play important parts in the inflammatory process: they encourage the secretion of cytokines and biomarkers, i.e., IL-1β, IL-2, IL-6, IL-8, TNF-α, IFN-γ and CRP, and chemokines such as CXCL11, CXCL16 and CCL5. They also fuel further inflammatory phenomena by stimulating other immunocompetent cells such as CTLs, NK cells and macrophages. The activity of pro-inflammatory cells and immune response mechanisms supports the formation of a chronic and persistent inflammatory state that can promote carcinogenesis [130,131,132].

The pro-inflammatory effects of certain diet components, i.e., saturated fatty acids, oxidized lipids, glucose and fructose, are believed to stimulate toll-like receptor (TLR) signalling via indirect binding to TLR2 and TLR4 receptors, and by activation of c-Jun N-terminal kinase-1 (JNK-1) and NF-κB-associated pathways, resulting in the activation of pro-inflammatory mediators. These have been proposed as the main molecular mechanisms triggering inflammatory changes in adipose tissue, inducing the transcription of pro-inflammatory genes giving rise to an inflammasome, and the release of several inflammatory cytokines [110,116,133,134,135,136,137]. The dietary components known to promote inflammation and obesity development also increase the activity of misfolded/unfolded protein response (UPR) elements, and promote their accumulation. This can also activate the JNK signalling pathway and stimulate persistent inflammation [138].

The free radicals that provoke oxidative stress, together with pro-inflammatory factors produced by immune cells, such as IL1β, IL-6, TNF-α, CRP, CXCL11, CXCL16 and CCL5, are also responsible for the activation of the Janus kinase (JAK) family: a group of intracellular, non-receptor tyrosine kinases that transduce cytokine-mediated signals via the signal transducer and activator of transcription 3 (JAK/STAT3) pathway [139,140,141]. The JAK/STAT3 pathway plays a key role in a number of cellular carcinogenic processes, including tumour cell growth and apoptosis: its components directly activate gene transcription of cytokines and pro-inflammatory molecules and mediate the expression of a variety of genes in response to cell stimuli [142,143,144].

Other pro-inflammatory diet components, including saturated fatty acids such as palmitic and stearic acid and oxidized lipids, cause oxidative stress. This is responsible for the activation of key signalling pathways through the phosphorylation of p38 mitogen-activated protein kinases (p38MAPK), extracellular signal-regulated kinases-1,2 (ERK1/2) and phosphoinositide 3-kinase/protein kinase B (PI3K/Akt), which play significant roles in lipotoxicity and result in excessively high transcription factor (NF-κB) and activator protein 1 (AP-1) activity. These favour cancer cell survival, and can also activate genes that facilitate cancer cell metastasis to other tissues [145,146,147].

Some of the pro-inflammatory dietary nutrients in food can act as antagonists of peroxisome proliferator-activated receptor gamma (PPARγ). Such antagonism inhibits the resolution of inflammatory phenomena and decreases the level and activity of adiponectin, resolvin and protectin; it can also reduce the potential of G protein-coupled receptor (GPR120) to inhibit NF-κB activation [110,135,148,149].

In summary, the well-known and widely considered unhealthy components of the modern pro-inflammatory diet pose a serious threat to the development of lifestyle diseases, including cancer. The signalling pathways presented above can intensify persistent chronic inflammation and promote the development and invasiveness of cancer. Indeed, carcinogenesis and tumour growth are known to be favoured by increased production of pro-inflammatory cytokines and chemokines (i.e., IL-1β, IL-6, CRP, IL-17, IL-23, CXCL11, CXCL16, CCL5), monocyte chemoattractant protein 1 (MCP-1) produced by adipocytes and macrophages, growth factors (i.e., VEGF, FGF2, HGF, IGF-1), adhesion molecules (i.e., VCAM, ICAM, E-cadherins, selectins), and matrix metalloproteinases (MMPs) including MMP-1, MMP-2, MMP-3 and MMP-9, which degrade various extracellular matrix proteins. These components of the tumour environment can play a major role in tumour cell behaviours such as elevated cell proliferation, migration (adhesion/dispersion), differentiation and angiogenesis. They can also promote invasiveness, cell migration and metastasis by influencing apoptosis and host defence.

**Figure 2:**
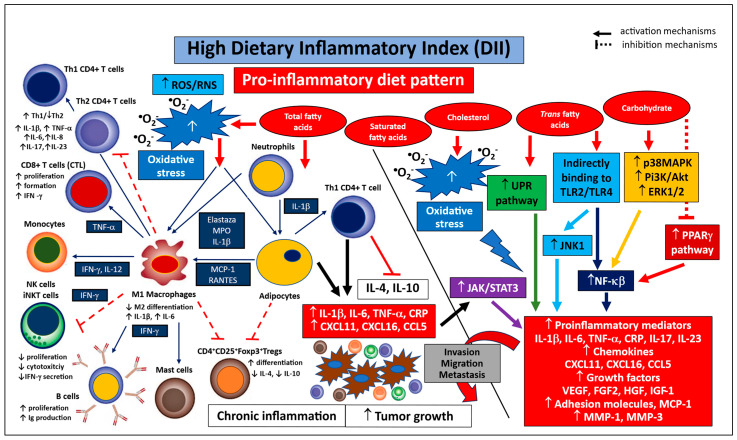
High dietary inflammatory index (DII), characterized by pro-inflammatory diet patterns promoting overall low-grade chronic inflammation (*metaflammation*—diet-induced inflammation) and potential carcinogenesis. The unhealthy diet components such as total fatty acids, saturated fatty acids (MUFA, PUFA), *trans* fatty acids, and cholesterol can activate adipocytes and macrophages and enhance oxidative stress in this inflammatory milieu. Adipocytes and macrophages can activate pro-inflammatory immune cells such as Th1 CD4^+^ T cells, CD8^+^ T cells (CTLs), Th17 T cells, monocytes, NK/iNKT cells, and mast cells and promote the dysfunction of anti-inflammatory cells, i.e., Th2 CD4^+^ T cells and regulatory T lymphocytes (CD4^+^CD25^+^Foxp3^+^ Tregs), leading to decreased production of anti-inflammatory cytokines such as IL-4, IL-10, and TGF-β. Free radicals, NO, ROS/RNS activate adipocytes that contribute to the attraction and shift of macrophages towards the pro-inflammatory M1 subpopulation. Neutrophils also contribute to the activation of adipocytes and TAMs through cathelicidins, which promote monocyte differentiation into M1-type macrophages and enhance neutrophil extracellular traps (NETs) phenomenon, directly stimulating macrophages to polarize towards M1 and activate the NLRP3 inflammasome, as well as promote the release of elastase, MPO, and IL-1β. Activation of the M1 subpopulation increases the proliferation and formation of pro-inflammatory immunocompetent cells. Increased proliferation and production of Ig antibodies by B cells also occur. The pro-inflammatory effects of diet components are related to the activation of pro-inflammatory mediators through stimulation of TLR signalling via indirect binding to TLR2 and TLR4 receptors, and by activation of JNK-1 and NF-κB-associated pathways, which support adipose tissue inflammation, inducing transcription of pro-inflammatory genes giving rise to an inflammasome, and releasing several inflammatory cytokines. An increased activity of the misfolded/unfolded UPR elements, which can also activate a JNK signalling pathway, is also observed. Oxidative stress induced by saturated fatty acids is also responsible for the activation of key signalling pathways such as p38MAPK, ERK1/2 and PI3K/Akt, which cause excessively high activity of NF-κB and AP-1. Some of the pro-inflammatory dietary nutrients can act as antagonists of PPARγ. Abbreviations: DII: dietary inflammatory index; MUFA: monounsaturated fatty acids; PUFA: polyunsaturated fatty acids; CTLs: CD8^+^ T cytotoxic lymphocytes; NK/iNKT: natural killer cells/invariant natural killer cells; TGF-β: transforming growth factor-beta; NO: nitric oxide; ROS/RNS: reactive oxygen species and reactive nitrogen species; MCP-1, CCL2: monocyte chemoattractant protein-1; RANTES, CCL5: regulated on activation, normal T cell ex-pressed and secreted; MPO: myeloperoxidase; MCP-1: monocyte chemotactic protein 1; TLRs: toll-like receptors; JNK-1: c-Jun N-terminal kinase-1; NF-κB: nuclear factor kappa-light-chain-enhancer of activated B cells; UPR: unfolded protein response (UPR); JAK/STAT3: the signal transducer and activator of transcription 3; p38MAPK: p38 mitogen-activated protein kinases; ERK1/2: extracellular signal-regulated kinases-1,2; PI3K/Akt: phosphoinositide 3-kinase/protein kinase B; AP-1: activator protein 1; PPARγ: peroxisome proliferator-activated receptor gamma (PPARγ); GPR120: G protein-coupled receptor. The figure constitutes the exclusive intellectual property of the author of the work. Primary sources used to create the Figure: [111,112,113,114,115,116,117,118,119,120,121,122,123,124,125,126,127,128,129,130,131,132,133,134,135,136,137,138,139,140,141,142,143,144,145,146,147,148,149].

The intracellular signalling pathways and mechanisms promoting persistent inflammation and pro-inflammatory immunocompetent cell populations associated with pro-inflammatory (high-DII) diet patterns are shown in Figure 2.

#### 1.2.2. Anti-Inflammatory Diet

A low dietary inflammatory index (DII), characterizing an anti-inflammatory diet pattern, was recognized as a practice parameter related to potent antioxidant, anti-inflammatory, anti-proliferative, and anti-tumourigenic foods. An anti-inflammatory diet, such as the Mediterranean diet (MD), macrobiotic diet, and plant-based (PB) diet, typically incorporates fruits, vegetables, whole grains, nuts, legumes, spices, herbs, and plant-based protein [64,150]. Such anti-inflammatory diets decrease the level of pro-inflammatory biomarkers such as IL-1β, IL-6, TNF-α and CRP and increase the concentration of anti-inflammatory cytokines IL-4 and IL-10, and TGF-β; they also prevent the development of chronic diseases, such as cancers, by inhibiting the pathways involved in maintaining a persistent inflammatory microenvironment. The components of anti-inflammatory diets not only directly suppress the persistent inflammatory response but also modulate the body’s metabolic processes activated by “smouldering” inflammation [150]. It is also worth emphasizing that the anti-inflammatory properties of these components derive from both their individual actions and synergistic interactions with other components. Research indicates that combining various ingredients enhances their anti-inflammatory effects [151].

Anti-inflammatory dietary components and foods are also known to influence the function of immunocompetent cells capable of suppressing chronic inflammation. These compounds include various carotenoids and polyphenols, such as phenolic acids, flavonoids (i.e., anthocyanins, flavones, flavanones, isoflavones, and flavanols), stilbenes or lignans, ω-3 fatty acids (primarily eicosapentaenoic acid and docosahexaenoic acid), dietary fiber, zinc, magnesium and vitamins C, E and D [150,152,153,154,155,156,157,158]. Such diets also reduce inflammation in the tumour environment. In the human body, their consumption results in the immune balance shifting toward Th2 CD4^+^ T cell activity, which is directly related to increased secretion of anti-inflammatory IL-4 and IL-10, and lower proliferation, formation, and activity of cytotoxic CD8^+^ T cells (CTLs). This mechanism is regulated by various inflammation suppressors such as transforming growth factor beta 1 (TGF-β1), prostaglandin E2 (PGE2), indoleamine-pyrrole 2,3-dioxygenase (IDO), inducible nitric oxide synthase (iNOS), and IL-1β. It is also characterised by increased differentiation and activity of typical cells that suppress the immune response, such as regulatory T cells (CD4^+^CD25^+^Foxp3^+^ Tregs), which also produce cytokines TGF-β, IL-4, IL-10, and IL-35. Tregs can produce granzyme B, which in turn can induce apoptosis of effector cells. This subpopulation also influences the induction of IDO and reverse signalling through direct interaction with dendritic cells (DCs). In addition, lower antigen presentation (oncoproteins from proto-oncogene/oncogene transcription) by qualified antigen-presenting cells (APCs), i.e., DCs, macrophages and B cells, can also inhibit the inflammatory response. Such inflammatory-inhibiting environments are also characterised by reduced differentiation and maturation of DCs, with lower antigen presentation, and decreased TNF-α and IL-12 production. A vital role in peripheral Treg generation and stability is played by vitamin A, converted to retinoic acid. Vitamin D is also needed to maintain cell homeostasis, and its deficiency is associated with reduced Treg cell numbers in tissues. Omega-3 fatty acids also promote the Treg subpopulation [150,152,153,154,155,156,157,158].

Furthermore, the balance between macrophages shifts from tumour-fighting M1 macrophages in favour of M2 cell activation and the production of pro-inflammatory IL-1β and IL-6. The main factors responsible for the inhibition of these cell functions are TGF-β1, IL-6, IL-10, PGE2, and IDO production. Omega-3 PUFA and polyphenols (e.g., curcumin, quercetin) act as natural activators of macrophages, changing their profile from M2 to M1 and alleviating adipose tissue inflammation [159,160]. Humoral response caused by the B cells is also inhibited, leading to decreased proliferation, Ig production, and their chemotactic function. For example, supplementing a diet with ω-3 fatty acids leads to increased B cell function and counteracts the negative effects of an inflammatory diet [161,162].

Moreover, a diet rich in antioxidants, trace elements, minerals and vitamins inhibits leukocyte chemotaxis; it has also been found to regulate endothelial dysfunction by downregulating the expression of cell adhesion molecules, such as VCAM, ICAM and E-selectin, in circulating immune cells and blocking the interaction between leukocytes and endothelial cells [153]. Furthermore, components such as polyphenols and flavonoids, curcumin, epigallocatechin-3-gallate (EGCG), resveratrol and genistein, as well as various sulfuric compounds (diallyl disulfide, DADS), may inhibit the function of various MMP genes (MMP-1, MMP-3, MMP-2 and MMP-9 and gelatinases; GLTs), and limit their expression [54,163,164].

The anticancer effects of anti-inflammatory diets are associated with their potential to inhibit oxidative stress. They may achieve this by reducing ROS and RNS production, and by doing so, the production of certain lymphokines (IL-1β, IL-6, TNF-α and CRP), and chemokines (CXCL11, CXCL16 and CCL5), resulting in immunosuppression [165,166,167,168,169,170]. Additionally, Zn supports antioxidative defence by activating cytosolic Zn/Cu superoxide dismutase, inhibiting NADPH oxidase and promoting the synthesis of cysteine-rich metallothionein [171]. In this environment, the production of other oxidative factors, such as iNOS, myeloperoxidase (MPO), and nitric oxide (NO), is also inhibited.

Antioxidant ω-3 fatty acids also exhibit synergistic activity with vitamin E; indeed, vitamin E has been found to further reduce the oxidative stress induced by these fatty acids [172,173]. Selenium and allyl mercaptan also appear to significantly downregulate the biosynthesis of cyclooxygenase (COX) and lipoxygenase (LOX), leading to reduced production of pro-inflammatory eicosanoids such as leukotrienes (LT) and prostaglandin 2 (PGE2) [166,169,174]. Some anti-inflammatory bioactive components (e.g., ω-3 fatty acids, fibre, polyphenols and vitamins A and E) reduce inflammation by inhibiting the activity of transcription factors such as NF-κB and activator protein 1 (AP-1); this results in the downregulation of certain pro-inflammatory cytokines (TNF-α, IL-6 and IL-1β) and the neutralization of ROS. It also downregulates the activation of lipopolysaccharides (LPS), endothelin-1, and other transcriptional factors such as c-Jun, STATs, the ERK/MAPK pathways, and protein kinase C (PKC). It has been proposed that dietary phytochemicals act by inhibiting the DNA-binding capacity of these enzymes [110,135,150,175,176].

Anti-inflammatory diet components can inhibit the function of NLRP3, a protein complex which serves as a crucial intracellular sensor of the innate immune system. Inhibition of NLRP3 prevents inflammasome formation and caspase-1 activation, which converts inactive cytokines into their active pro-inflammatory forms (IL-1β, IL-18) responsible for chronic inflammation. For example, ω-3 fatty acids (EPA and DHA) inhibit NLRP3 function by inter alia affecting GPR120 receptors. Polyphenols and antioxidants such as curcumin, resveratrol and EGCG block key signalling pathways, including the NF-κB pathway, essential for inflammasome function [177,178]. However, they also block pyroptosis, or rapid cell death; this may be of value in anti-cancer therapy, as it prevents the elimination of pro-inflammatory factors and inhibits the activity of immunocompetent cells [150]. T-lymphocytes can not only undergo pyroptosis but also use it to attack tumour target cells. Under such conditions, cytotoxic T cells (CD8^+^ CLTs) release granzymes that can directly destroy gasdermins (GSDMB or GSDME) in the cancer cell. The destruction of the cancer cell releases alarm signals (DAMPs) that attract and stimulate more immune cells. CD4^+^ lymphocytes can also undergo pyroptosis following activation of the NLRP3 inflammasome stimulated by calcium influx [179,180,181].

Anti-inflammatory dietary components may also regulate the action of nuclear factor erythroid 2-related factor 2 (Nrf2), a key protein that activates various antioxidant systems and the detoxification response [182,183]. Studies suggest that Nrf2 activates over 250 genes responsible for protecting cells from oxidative stress and high inflammatory status in response to a range of oxidative stressors, such as ROS, RNS, NO, PGLs, and oxidized low-density lipoproteins (LDLox). Oxidative stress, persistent inflammation, or exposure to anti-inflammatory bioactive compounds result in the activation of Nrf2 cysteines; these interact with the Kelch-like ECH-associated protein 1 (Keap1) and disrupt an adaptor component of the Cul3 complex. Upon activation, Nrf2 enters the cell nucleus, where it forms a complex with Maf proteins and the antioxidant response element (ARE). Phase II antioxidant enzymes are activated by inter alia glycates and indol-3-carbinol; they then induce enzymatic antioxidants such as superoxide dismutase (SOD) and catalase (CAT), which neutralize free radicals. They also silence the production of pro-inflammatory cytokines by inhibiting the activity of NF-κB. Nrf2 also supports DNA repair, supports genome stability and cellular energy metabolism, and exhibits antiproliferative effects [110,175]. Similarly, zinc and magnesium have also been found to support DNA repair [166,168].

Importantly, dietary components containing antioxidant factors such as polyphenols (e.g., epigallocatechin gallate EGCG, resveratrol), curcumin, flavonoids (e.g., genistein), minerals (e.g., selenium), organosulfur constituents (allyl mercaptan), and vitamins (e.g., folate) can also modulate the pro-apoptotic response and reverse cell cycle checkpoint function. Moreover, some food components rich in fats and carbohydrates may increase the expression of suppressor genes and inhibit proto-oncogenes through various epigenetic mechanisms, e.g., DNA methylation driven by DNA methyltransferases (DNMTs), histone modifications regulated by histone deacetylase (HDAC) or histone acetyltransferase (HAT), gene regulation by non-coding RNA (nc-RNA), i.e., micro-RNAs (miRNAs) or long non-coding RNAs (lncRNAs). These mechanisms may inhibit cancer formation [166,167,184,185,186,187,188].

Another important aspect of an anti-inflammatory diet is the impact of polyphenols (e.g., flavonoids, hydroxytyrosol, oleuropein, and resveratrol), unsaturated fatty acids, and ω-3 fatty acids on other key cellular mechanisms. For example, these components can inhibit the formation of advanced glycation end-products (AGEs) produced during frying, grilling, and baking. This can reduce the activation of AGE receptors (RAGE), thus downregulating chronic inflammation and oxidative stress [189,190]. These anti-inflammatory compounds also lower TLR (TLR-2, TLR-4, TLR-5) pathway signalling and inhibit NF-κB activity, thus reducing the release of pro-inflammatory cytokines [191,192]. They also lower lipogenic gene expression and increase the production of lipid mediators such as E- and D-resolvins; they also promote the incorporation of ω-3 fatty acids over arachidonic acid (AA) in the phospholipids of immune cell membranes (e.g., monocytes, macrophages), leading to the altered formation of ω3- and ω5-series of eicosanoids. These reduce persistent inflammatory status and exert potent anti-inflammatory actions in inter alia neutrophils, macrophages and T-cells [190,193]. They also have a significant prebiotic effect on gut bacteria, increasing their diversity [194]. It has been observed that decreased microbial diversity and increased gram-negative anaerobic anaerobes/microaerophile bacteria may contribute to the development of oesophageal cancer by disrupting the host immune response. The activation of the LPS-TLR4-NF-κB pathway may contribute to inflammation and malignant transformation [195,196].

In summary, certain bioactive components of an anti-inflammatory diet, found in vegetables and fruits, described as “green chemoprevention products,” may reduce the risk of developing lifestyle diseases, including cancer. The intracellular and intercellular mechanisms described above, occurring in the tumour matrix, clearly indicate that natural foods included in an anti-inflammatory diet may help inhibit tumour growth. This is facilitated by, among others, enhanced immunosuppression mechanisms, greater inhibition of chronic inflammation, reduced pro-inflammatory mediator secretion, increased cell adhesion and reduced cell migration and invasion. Such a diet can also inhibit the function of tumour MMPs, reduce cell growth through inhibition of growth factor synthesis, and enhance proapoptotic, antiproliferative, antiangiogenic, and anti-tumour activity. It can also induce genetic changes that promote protection from DNA damage and reduce the transcription and activity of proto-oncogenes.

**Figure 3:**
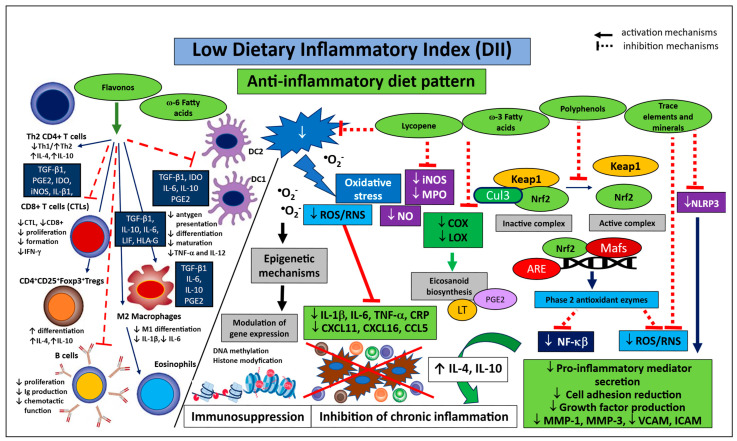
Low dietary inflammatory index (DII), which characterizes an anti-inflammatory diet pattern, is related to antioxidant and anti-inflammatory properties. The function of relevant subpopulations of immunocompetent cells is associated with suppressing chronic inflammation. During smouldering inflammatory process, a shift in the immune balance toward Th2 CD4^+^ T cell activity occurs, which is directly related to the production of inhibitory cytokines IL-4 and IL-10, and a simultaneous inhibition of the proliferation, formation, and activity of CD8^+^ T cells (CTLs). There is also increased activity of T cells that suppress the immune response, such as CD4^+^CD25^+^Foxp3^+^Tregs. Inhibition of the inflammatory response is also directly related to the reduction of antigen (oncogene) presentation by qualified APCs, i.e., DCs. Furthermore, the activity of anti-inflammatory and anti-tumorigenic M1 macrophages decreases in favour of M2 cell activation. The humoral response caused by the inhibition of B cells is also inhibited. The anticancer effect of an anti-inflammatory diet relates to the inhibition of oxidative stress, reduced production of ROS and RNS, and consequently, reduced production of lymphokines such as IL-1β, IL-6, TNF-α, CRP, and the chemokines CXCL11, CXCL16, and CCL5, leading to immunosuppression. Anti-inflammatory components inhibit inflammation in the tumour milieu by regulating transcription factors such as NF-κB, thereby leading to reduced production of pro-inflammatory cytokines, i.e., TNF-α, IL-6 and IL-1β. Also, inhibition of the function of NLRP3 leads to the inability to form the inflammasome and the inability to activate caspase-1. Chronic inflammation may also be related to the activity of nuclear factor Nrf2, a key protein that acts as an activator of the body’s antioxidant and detoxification response. Nrf2 is a transcription factor that activates genes responsible for protecting cells from oxidative stress and inflammatory status. Under conditions of oxidative stress and persistent inflammation, Nrf2, Keap1, and an adaptor component of the Cul3 complex are disrupted. Upon activation, Nrf2 enters the cell nucleus, where it binds to form a complex with Maf proteins and ARE. Activated phase II antioxidant enzymes inhibit the formation of ROS and RNS, inducing enzymes such as SOD and catalase, which neutralize free radicals. They inhibit the activity of the transcription factor NF-kB, thus silencing the production of proinflammatory cytokines. Some food components could modify or alter key gene expression through epigenetic mechanisms, i.e., DNA methylation, catalysed by enzymes known as DNMTs, HDAC or HAT, and modulation and regulation of genetic expression by nc-RNA, i.e., miRNAs. Abbreviations: DII: dietary inflammatory index; CTLs: CD8^+^ T cytotoxic lymphocytes; APCs: antigen-presenting cells; DCs: dendritic cells; ROS: reactive oxygen species; RNS: reactive nitrogen species; COX: cyclooxygenase; LOX: lipoxygenase; LTs: leukotrienes; PGE2: prostaglandin 2; NF-κB: nuclear factor kappa-B; NLRP3: nucleotide-binding domain, leucine-rich-containing family, pyrindomain-containing-3; Nrf2: nuclear factor erythroid 2-related factor 2; Keap1: Kelch-like ECH-associated protein 1; ARE: antioxidant response element; SOD: superoxide dismutase; DNMTs: DNA methyltransferases; HDAC: histone modifications regulated by histone deacetylase; HAT: histone acetyltransferase; nc-RNA: non-coding RNA; miRNAs: micro-RNAs. The figure constitutes the exclusive intellectual property of the author of the work. Primary sources used to create the Figure: [150,151,152,153,154,155,156,157,158,159,160,161,162,163,164,165,166,167,168,169,170,171,172,173,174,175,176,177,178,179,180,181,182,183,184,185,186,187,188,189,190,191,192,193].

The mechanisms and compounds inhibiting persistent inflammation are summarised in Figure 3. The figure also indicates the share of key subpopulations of immunocompetent cells related to anti-inflammatory diets characterized by low dietary inflammatory index (DII).

In summary, the subtitles of the article in this section, devoted to regulatory mechanisms, present signalling pathways that represent proposed interpretations of the pathways stimulated/inhibited by inflammation during general carcinogenesis. Importantly, the pathways regulating the function of immunocompetent cells under the influence of inflammation (pro- and anti-inflammatory environments) are proven and widely accepted in head and neck cancers (HNSCC). The remaining proposed pathways, still under discussion and interpretation by many authors, are typical for inflammation and obesity and represent well-understood mechanisms in general cancer biology and constitute extrapolated pathways. Therefore, further research on the impact of diet and nutritional behaviour and habits on intracellular pathways requires extensive experimental and clinical research.

## 2. Materials and Methods

A comprehensive literature search was conducted on the relationship between DII and E-DII and the risk of developing cancers of the upper aerodigestive tract (UADT). The search also addressed the influence of DII and E-DII on systemic inflammatory status and its role in the pathogenesis of HNSCC. It also included an analysis of the key pro- and anti-inflammatory mechanisms of diet and diet-dependent signalling that may influence HNSCC carcinogenesis.

The searched corpus encompassed a wide range of key opinion-forming systematic reviews, as well as molecular, observational, cross-sectional and case-control studies in humans. The final search (conducted on 31 May 2026) included the most valuable and highest-rated peer-reviewed articles published from the last decade (January 2015 to May 2026), all of which are accessible via PubMed/EMBASE/Web of Science.

### Literature Search

The articles were analysed in accordance with the Systematic Reviews and Meta-Analyses extension for Scoping Reviews (PRISMA 2020) guidelines, also taking into account systematic reviews [197]. The databases were searched using the following keywords: “dietary inflammatory index or DII”, “energy-adjusted dietary inflammatory index or E-DII”, “inflammatory dietary pattern”, “dietary inflammation score”, “anti- and pro-inflammatory diet”, “immune-inflammation”, “head and neck neoplasm or HNSCC”, “squamous cell carcinoma of head and neck”, “head and neck cancer”, “head and neck carcinoma”, “upper aerodigestive tract cancers or UADT”, “oesophageal cancer”, “oral cancer”, “nasopharyngeal cancer”, “oropharyngeal cancer”, “laryngeal cancer”. An additional record by cross-references was identified. The search also included tumours of the oesophagus and nasopharyngeal region, such as squamous cell carcinomas: these demonstrate a similar histology to common head and neck squamous cell carcinomas (HNSCC) and represent suitable comparisons of data regarding the effect of DII/E-DII on site-specific head and neck cancers.

Specific details on the search strategies are detailed in Table 1.

The following inclusion criteria were implemented: (a) written in English language; (b) studies based on humans; (c) cohort studies, observational studies, case-control studies examining the association between DII/E-DII score and HNSCC risk; (d) key opinion-forming systematic reviews; (e) DII value was computed at baseline with continuous or categorical DII (the highest vs. lowest or higher vs. lower DII category); (f) studies that provided original risk ratio (RR), hazard ratio (HR) or odds ratio (OR); (g) patients with confirmed diagnosis of HNSCC, including ESCC.

The following exclusion criteria were applied: (a) unpublished articles or conference proceedings; (b) case reports, case records, and letters to the editor; (c) studies where the patient’s diagnosis is uncertain (e.g., no histopathological confirmation); (d) abstracts. The papers that merely reported an association between HNSCC carcinogenesis and DII/E-DII score were also excluded.

**Scheme 1:**
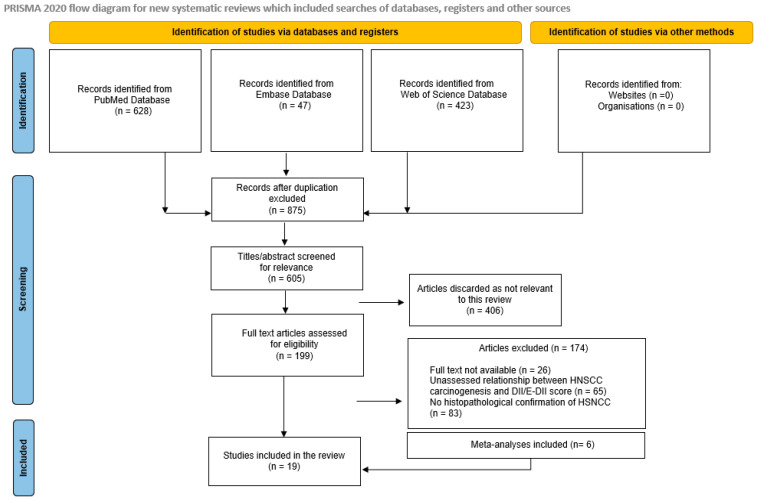
PRISMA 2020 flow diagram for systematic review which included searches of databases, registers and other sources.

This work discusses the obtained issues in detail, in order of increasing clinical credibility. Relevant information regarding the objectives, methods, results, and conclusions was collected from the extracted articles. A total of 199 articles were recognized after the literature search, and of these, 19 pertinent articles were selected for inclusion in the review (Scheme 1).

## 3. Results

Epidemiological, clinical and molecular data suggest that a pro-inflammatory diet characterized by a high DII/E-DII index may play a role in the pathogenesis and progression of human cancers; however, more precise data from the last decade on head and neck cancers are limited and even divergent. The relationship between prior dietary DII/E-DII parameters and HNSCC has formed the basis of over a dozen observational studies. The most recently published original articles, systematic reviews, and meta-analyses consistently indicate that many HNSCC patients demonstrate high or very high DII/E-DII scores, and this was quantitatively associated with the incidence of various head and neck cancers [55,57,98,99,198,199,200,201,202,203,204,205]. In addition, obesity induced by a diet rich in pro-inflammatory factors, characteristic of the Western and fast-food diets, may increase the risk of chronic inflammation and a lower final DII/E-DII index value. Furthermore, inflammation can be promoted by food insecurity and malnutrition, and generally poor nutrition, caused by low socioeconomic status, impaired absorption of healthy dietary components, and a deficiency of dietary nutrients and supplements rich in anti-inflammatory compounds.

However, to date there has been no clear evidence of a causal relationship between the DII/E-DII indicator scale and the pathogenesis of head and neck cancer. Importantly, clinical analyses have revealed discrepancies in the reported data and final conclusions regarding this relationship [55,56,57].

### 3.1. HNSCC Risk in Relation to DII/E-DII Score in Case-Control Studies

A body of literature over the past decade supports the hypothesis that high DII/E-DII levels are associated with a higher risk of chronic inflammation in the course of neoplastic disease, and an increase in the risk of HNSCC [55,57,98,99,198,199,200,201,202,203,204,205]. It has been proposed that anti-inflammatory nutrients present in unprocessed or minimally preserved or modified foods rich in various antioxidant compounds may fulfil a protective function by inhibiting angiogenesis, cellular proliferation and inflammation, and promoting apoptosis and cellular differentiation. These compounds are believed to be supported by marine ω-3 PUFAs, fiber and natural spices, as well as by vitamins and some trace elements such as those present in the Mediterranean (MD), macrobiotic and plant-based (PB) diets. Many pre- and clinical studies have found high doses of anti-inflammatory factors to inhibit cell cycle progression and tumour growth, and induce apoptosis, in cancer cells [166,167,184,185,186,187,188]. Numerous clinical studies have found patients with UADT cancers to be frequently deficient in anti-inflammatory nutrients. However, head and neck cancer (HNC) has been relatively rarely studied in relation to DII/E-DII score, with only a few case-control studies published to date [55,57,98,99,198,199,200,201,202,203,204,205].

These findings have been confirmed in a recent Iranian case-control study of 879 consecutive patients with a diagnosis of neoplastic HNSCC disease [57], in which the authors found a more pro-inflammatory diet and higher DII score to be associated with a greater risk of all HNSCC studied (OR = 1.31, p*_trend_* = 0.013). Regarding the site of HNSCC neoplasm, the authors found a higher DII score to be associated with a higher tumour risk index for lip and oral cancer (OR = 1.56, p*_trend_* = 0.004) and pharyngeal cancer (OR = 2.08, p*_trend_* = 0.02). Importantly, no such association was confirmed for laryngeal cancer. An interaction was also confirmed between tobacco smoking and DII on the risk of HNSCC. Patients who used tobacco had higher DII scores and were more likely to be in the third tertile and at a higher risk of HNSCC; in comparison, those who did not use tobacco were most often in the first tertile (OR = 2.52, p*_interaction_* = 0.03).

A recently published work by Bao et al. [198] analysed a group of 295 patients with oral cavity cancer (OSCC). The authors indicate that the risk of OSCC was higher in patients in the fourth, i.e., highest quartile compared to those in the lowest quartile. This risk increased 2.56-fold (OR = 2.56, p*_trend_* < 0.001) after adjusting for selected covariates. Subsequently, when E-DII was calculated as a continuous variable, the risk of OSCC increased by 3% (OR*_continuous_* = 1.03, p*_trend_* < 0.001) for a one-unit increase. Interestingly, stronger associations were noted between E-DII and OSCC among male subjects, those aged > 60 years, never-smoking subjects and alcohol drinkers, and individuals with poor hygiene.

Similar results were obtained by Secchi et al. [99] in a small group of 27 OSCC patients. The findings indicate a positive relationship between a higher E-DII index score and a higher OSCC risk close to 18.5-fold (OR = 18.46, p*_trend_* = 0.003) after adjusting for alcohol and tobacco consumption. In addition, the risk of developing a malignant tumour was 69% higher when taking the E-DII score into account (OR = 1.69, p*_trend_* = 0.0029).

Findings obtained as part of the Carolina Head and Neck Cancer Epidemiology (CHANCE) study [98] (1389 patients) indicated significantly higher DII scores in HNSCC cancerous lesions when comparing the results within quartiles. The highest E-DII score was associated with an increased risk of all HNSCC compared to the lowest E-DII quartile (OR = 2.91, p*_trend_* < 0.001). The risk varied depending on the tumour site: it increased 3.5-fold for laryngeal cancer (OR = 3.49, p*_trend_* < 0.001), 2.92-fold for oropharynx (OR = 2.92, p*_trend_* < 0.001), and 2.47-fold for oral cavity cancer (OR = 2.47, p*_trend_* = 0.01).

Abe et al. [55] obtained similar findings in a study assessing the predictive value of DII score in 1028 patients with upper aerodigestive tract cancers (UADT). The cohort included individuals with various tumour sites such as oesophageal cancer (ESCC) and HNCs with various subsites. The results indicated the DII level was related to a 73% higher risk of UADT and ESCC analysed together, and a 92% greater risk of HNSCC after adjusting for selected covariates (OR = 1.73, *p* < 0.001; OR = 1.92, *p* < 0.001, respectively). Furthermore, regarding HNSCC subsites, the greatest increase in risk was observed for HPSCC (OR = 4.05, *p* = 0.04) and OSCC (OR = 2.38, *p* < 0.001). However, no correlation was confirmed between DII score and LSCC, NPSCC, and OPSCC incidence. Importantly, variables such as tobacco smoking and alcohol consumption also influenced the results: the risk of UATD initiation was significantly higher in ever smokers (by 48%; OR = 1.48, p*_trend_* = 0.007) and ever drinkers (59%; OR = 1.59, p*_trend_* = 0.003).

Similarly, Tang et al. [199] also found a higher E-DII score to be associated with oesophageal cancer in Chinese patients. The study showed that the risk of ESCC was related to the categorical E-DII factor, and specifically, the highest DII quartile was associated with a 2.55-times increased risk of ESCC compared to the lowest DII quartile (OR = 2.55, p*_trend_* < 0.001). In addition, a significant interaction was noted for selected variables: tobacco smoking was associated with a 59% higher risk of ESCC (OR = 1.59, p*_trend_* = 0.08) and ethnic minority group with 86% (OR = 1.86, p*_trend_* = 0.002).

A case-control study by Shivappa et al. based on a FFQ also identified a strong positive relationship between oral and pharyngeal cancer and E-DII score in a study group of 946 patients [200]. The findings indicate that the highest E-DII score was associated with a 1.8-fold higher risk of OSCC and PSCC combined (OR = 1.80, p*_trend_* < 0.0001). Similarly, in the group with the highest E-DII scores, the risk of developing cancer increased by 2.08-fold for oral cancers assessed individually (OR = 2.08, p*_trend_* < 0.0001) and 1.6-fold for pharyngeal cancers (OR = 1.6, p*_trend_* < 0.0001), compared to those with the lowest scores. Interestingly, similar relationships were noted for subsites of pharyngeal cancer, with the same OR results noted for OPSCC (OR = 1.60) and HPSCC (OR = 1.64), respectively. A 1.17-fold higher risk of OSCC and PSCC was also observed after adjusting for the dose-response association. The findings were influenced by the inclusion of selected variables. A significant association between highest and lowest DII was observed for patients aged ≥60 years, women, and, importantly, for tobacco smokers (OR = 4.43, RERI 1.31, *p* = 0.01) and alcohol drinkers (OR = 5.87, RERI 2.43, *p* < 0.001) compared to never smokers and never drinkers, respectively.

Lu et al. [201] also analysed the relationship between the risk of oesophageal cancer and E-DII score. An E-DII score in the higher quartile was found to be associated with up to 4.35 times greater risk of ESCC (OR = 4.35, p*_trend_* = 0.0001). Furthermore, a high E-DII score was associated with a high BMI (≥25 kg/m^2^) (OR = 6.60, p*_trend_* = 0.006).

In addition, a case-control study by Shivappa et al. including 198 patients with nasopharyngeal cancer (NPSCC) found E-DII score to correlate with a greater than 60% risk of neoplastic disease when adjusted for energy intake and other selected variables (OR = 1.64, p*_trend_* = 0.01) [202]. The authors also confirmed that a higher E-DII score was associated with a nearly 20% increased risk of NPSCC.

A study on an Italian population (n = 460 cases) by Shivappa et al. [203] examined the relationship between the risk of laryngeal cancer (LSCC) and the E-DII index. It was found that the risk of LSCC differed by more than 3.30-fold between the extreme quartiles (OR = 3.30, p*_trend_* < 0.0001) and 1.27-fold when E-DII was included as a continuous variable (OR*_continues_* = 1.27 for a 1-unit increment in the DII, p*_linearity_* < 0.001). When the results were stratified based on tobacco smoking, alcohol consumption, age, and BMI, it was found that the risk of LSCC increased by 6.64 times for current smokers compared to never/ex-smokers. In addition, the risk of LSCC was significantly (5.82 times) greater for heavy drinkers compared to never-to-moderate drinkers.

Two other studies by Shivappa et al. [204,205] have also confirmed that a high E-DII score may lead to a greater risk of oesophageal cancer (ESCC), indicating that this parameter may be of value for predicting the onset of carcinogenesis in the upper aerodigestive tract. In the first study E-DII score was associated with a significant increase, i.e., of nearly 724%, in the risk of ESCC after multivariate adjustment. Similarly, analysis of continuous E-DII also showed a significant increase in the risk of ESCC, i.e., by over 350%, in the multivariate analysis, with a one-unit increase corresponding to nearly 16% of its range [204]. In the second study, higher ESCC risk was found to correlate with a higher E-DII score; it was found to increase the possibility of developing a malignant tumour by 2.47-fold in multivariable analysis. In addition, the risk of ESCC grew to 1.39-fold when using the continuous E-DII. Interestingly, no significant interaction was noted with sex, alcohol drinking, or tobacco smoking [205].

In summary, all these studies except one [55] indicate that E-DII score is a good epidemiological indicator for estimating the risk of head and neck squamous cell cancer. The data clearly support that eating a healthy anti-inflammatory diet, i.e., with a low DII/E-DII score, is associated with a lower risk of developing HSNCC than a pro-inflammatory diet, i.e., with a high DII/E-DII score.

Table 2 summarizes the characteristics of the observational studies included in the review and the data collected from them.

### 3.2. The Results of HNSCC Risk in Relation to DII/E-DII Score in Meta-Analyses

The present study includes all meta-analyses on the topic published in the last decade, as these remain the most valuable, reliable and definitive bases for analysis. Their conclusions were derived from the risk estimates for HNSCC types derived from the original studies given above, i.e., oral cancer (OSCC), pharyngeal cancer (PSCC), laryngeal cancer (LSCC), and oesophageal cancer (ESCC). The analysis considers inter alia heterogeneity among studies, research quality, sample size, methodology (FFQ items and DII components), and geographic region, in relation to the DII/E-DII score. Most importantly, most studies indicate that patients with significantly higher DII/E-DII scores had a higher risk of carcinogenesis in the head and neck region [47,53,54,59,206,207].

For example, Shrivastava et al. [59] propose that E-DII score may be used as a predictor for OSCC and PSCC development. Their study, encompassing five case-control studies covering the years 2017–2020 in a population of 2621 patients, showed that the risk of oral cancer is 128% higher (OR = 2.28, *p* < 0.00001), and of pharyngeal cancer 165% higher (OR = 2.65, *p* < 0.00001) in patients with the highest DII compared to those with the lowest DII. Similarly, the risk of combined OSCC and PSCC together was 132% (OR = 2.32, *p* < 0.00001) higher in patients with the highest DII score compared to those with the lowest. Interestingly, the studies were characterized by a low or extremely low heterogeneity index (*I*^2^ = 38%, *I*^2^ = 0%, and *I*^2^ = 28%, respectively).

The same group of case-control research was analysed by Luo et al. [206] considering DII scores in a cohort of 1278 OSCC patients with oral cancer. The risk of OSCC was 2.35 times higher in patients with the highest categorical DII score compared to the lowest and 1.79-fold higher in individuals with higher DII compared to lower. Regarding the dose-response association between DII and OSCC, a 1-unit increment in DII was associated with a 1.17-fold increase in cancer risk; however, significant heterogeneity was noted among included studies (*I*^2^ = 86.1%). The researchers attribute these *I*^2^ values to the use of a short 47-item FFQ by Abe ta et al. [55] and to the small sample size (15 OSCC cases) in Secchi et al. [99]. The researchers found no evidence of a nonlinear association of DII with oral cancer.

Similar data were obtained from an analysis of eight case-control studies [54]. The researchers found that among 2556 cases with upper aerodigestive tract (UADT) cancer, the risk of neoplastic disease was 2.07-fold higher when comparing the highest DII values with the lowest. A similar association was also confirmed in the higher and lower DII score groups, with a 1.53-fold increase in the risk of UADT. The data also showed a positive relationship between dose-response association and OSCC risk. The calculated RR for a 1-unit increment in the DII score was 1.18; however, with significant heterogeneity among studies included (*I*^2^ = 82.1%). Interestingly, the authors also found the risk of head and neck carcinogenesis to increase with age and that it was 2.27 times higher among patients >60 years old. The researchers conclude that the risk of UADT cancers was nearly 180% greater among studies with higher quality than in those with lower quality.

Hua et al. [53] examined the relationship between the risk of UADT and DII score in an analysis of nine case-control studies from the period 2015–2018, comprising 7202 patients. The study calculated the risk of carcinogenesis not only for the entire UADT cancer group but also for specific tumour subsites. The analysis showed a 127% increase in UADT risk for the highest DII score category compared to the lowest. Similarly, a meta-analysis found the highest DII score to be associated with significantly greater risk than the lowest DII score for the various cancer types: oesophageal cancer (2.53-fold), oral cavity cancer (2.23-fold), and pharyngeal cancer (2.02-fold). Interestingly, when stratified by geographical region, the highest risk of UADT was observed in the USA, where the highest DII score was associated with a 201% increase in cancer risk (OR = 3.01); in Europe, with a 119% increase (OR = 2.19); the lowest risk (111%) was recorded in Asia (OR = 2.11).

In a meta-analysis of 1961 patients with oesophageal cancer (ESCC), Chen et al. [47] found the patients with the highest DII score to have a 2.54-times higher risk of cancer than those with the lowest DII score (OR = 2.54). However, the authors pointed to significant heterogeneity in the selected studies, which could result from the published year of the analyses and the number of components included in the FFQs. Indeed, publications from 2018 were characterized by the highest heterogeneity (*I*^2^ = 63.3%), and the lowest heterogeneity in studies from 2015 (*I*^2^ = 35.7%). Moreover, there was significant heterogeneity in studies estimating < 30 FFQ components (*I*^2^ = 63.8%), while no heterogeneity was observed in studies with ≥30 components (*I*^2^ = 25.1%). The researchers suggest that Abe et al. [55] may represent a source of heterogeneity in the entire meta-analysis.

An analysis of five case-control studies comprising 1167 HNSCC cases by Jayedi et al. [207] found a one-unit increment in the DII score to be associated with a 24% higher risk of oesophageal cancer (OR = 1.24), a 27% higher risk of laryngeal cancer (OR = 1.27) and a 9% higher risk of nasopharyngeal cancer (OR = 1.09).

Hence, a healthy anti-inflammatory diet described by a lowest/lower DII/E-DII score, mainly containing fruits and vegetables, may reduce the risk of developing squamous cell carcinoma of the head and neck region. In contrast to the pro-inflammatory diet, i.e., with highest/higher DII/E-DII values, such a diet may constitute a preventive dietary pattern and play a valuable role in HNSCC risk control. However, this analysis has important limitations, which will be given in the Discussion.

Table 3 summarizes the selected meta-analyses from the last decade found in the review and the data collected from them.

## 4. Discussion

The dietary inflammatory index (DII), priori-determined pattern, is an innovative and proven quantitative tool that analyses the inflammatory potential of an individual’s diet. Its use allows for an objective assessment of the impact of diet on inflammatory status and of the risk of developing lifestyle diseases such as neoplastic diseases [37]. This narrative review presents the latest research regarding the use of the DII in predicting the risk of cancers of the head and neck (HNC). The study also includes its modification, viz. the energy-adjusted dietary inflammatory index (E-DII): a parameter that considers total energy intake when assessing the inflammatory potential of a diet [85]. Therefore, this work represents a valuable compendium of knowledge on the etiopathogenesis of HNSCC, DII/E-DII scores and diet-induced inflammation, and includes a summary of all research regarding HNSCC risk estimation based on DII/E-DII indicators up to 31 May 2026.

Global data on the occurrence of HNSCC suggests that pro- or anti-inflammatory diet components, indicated by DII/E-DII score, and specific inflammation-inducing dietary patterns play a key role in determining the risk of head and neck cancer (HNC), including oesophageal cancer. These findings appear to remain true for cancer site, ethnicity, age, sex, number of DII components and follow-up duration in most publications. Indeed, inflammatory components from the daily diet may play a role in the initiation of HNSCC carcinogenesis and its further stages by participating in the formation of a persistent inflammatory tumour microenvironment (TME) [105,106,107,108,109,110,111,112,113,114,115,116,117]. There is clear evidence that healthy eating habits and the consumption of an anti-inflammatory dietary programme rich in fruits, vegetables, whole grains, fish, olive oil and ω-3 and ω-6 fatty acids can play a protective role and may prevent the development of HNSCC [150,152,153,154,155,156,157,158]. Conversely, following a pro-inflammatory diet profile, including fast food, processed nutrients, snacks and animal products, has been shown to promote their onset [53,97,98,99]. Pooled data from thirteen observational studies and six meta-analyses published by May 31, 2026, found a pro-inflammatory diet characterized by the highest/higher DII and E-DII ratios to be a significant indicator of an increased risk of developing HNSCC tumours in most studies [47,53,54,55,57,59,195,196,197]. Indeed, the highest DII/E-DII categories may even be associated with 102–165% greater risk of HNCs, compared with the lowest scores, and the higher categories with a 53–79% greater risk than the lower scores [54,59,206]. Regarding linear dose-response analyses, a one-unit increment in DII/E-DII score was related to an increase of 17–18% in risk of upper aerodigestive cancers [54,206]. These observations suggest that, in addition to stopping smoking and controlling alcohol consumption, further efforts should be made to promote protective anti-inflammatory dietary habits, as this too represents a significant preventative measure that has a real impact on the development of HNSCC.

The overwhelming majority of publications and meta-analyses indicate that consuming fruits and vegetables, fatty fish, unprocessed grain products, and low-fat meats has a beneficial protective effect against the risk of head, neck, and oesophageal cancers. Interestingly, a meta-analysis by Pavia et al. [208] clearly demonstrated that each daily serving of fruits and vegetables can reduce the overall risk of oral cancer by 50%. The protective effect of an anti-inflammatory diet rich in fruits and vegetables has been attributed to the properties of numerous dietary anticancer substances, such as carotenoids, glucosinolates, isothiocyanates, protease inhibitors, phenols, plant sterols, flavonoids, polyphenols, selenium, and dietary fibre. Their anti-inflammatory properties, responsible for the low DII and E-DII ratio, exhibit immunosuppressive and anti-cancer effects, either individually or synergistically. They also inhibit low-grade inflammation by influencing the function of anti-inflammatory subpopulations of immunocompetent cells such as Th2 CD4^+^ T cells, CD4^+^CD25^+^Foxp3^+^Tregs and anti-tumourigenic M2 macrophages; they also regulate key cell signalling pathways by influencing transcription factors such as NF-κB and Nrf2, the function of the NLRP3 and PPARγ pathways, inhibit oxidative stress, and reduce production of ROS and RNS. As a result, they also reduce production of pro-inflammatory lymphokines such as IL-1β, IL-6, TNF-α, CRP, and the chemokines CXCL11, CXCL16, and CCL5, and increase the production of immunosuppressive cytokines such as IL-4 and IL-10. Anti-inflammatory dietary compounds also inhibit the cell cycle and angiogenesis, regulate apoptosis and influence epigenetic mechanisms; also, by acting as antioxidants and inducers of detoxification enzymes, they inhibit the formation of other carcinogens and increase the production of anticancer substances. They thus limit the ability of transformed cells to proliferate and alter cellular metabolism [150,152,153,154,155,156,157,158,159,160,161,162,163,164].

Conversely, a pro-inflammatory diet characterized by high DII and E-DII indices activates several immune cells and cellular pathways that promote cancer development. Such diets may be rich in pro-inflammatory foods, such as fast food, seed, butter and animal products, with elevated levels of total fatty acids, saturated fatty acids (MUFA, PUFA), trans fatty acids and cholesterol. These components promote the induction and maintenance of a “smouldering” state of metaflammation, i.e., diet-induced inflammation. Pro-inflammatory dietary patterns promote the induction of immunocompetent pro-inflammatory cells such as adipocytes and M1-phenotype macrophages, which are able to activate Th1 CD4^+^ T cells, CD8^+^ T cells (CTLs), Th17 T cells, NK/iNKT cells, monocytes and mast cells. They also induce pro-inflammatory pathways such as TLR signalling, JNK-1 and NF-κB-associated pathways, and cytokine-mediated signalling via the JAK/STAT3 pathway and p38MAPK, ERK1/2, and PI3K/Akt. These pathways drive the production of IFN-γ, TNF-α, IL-1β, IL-6, IL-12, and MCP-1, and promote tumourigenesis and subsequent cancer development [165,166,167,168,169,170]. In addition, preparing snacks with high fat content and curing meat, characteristic of the pro-inflammatory diet, result in the formation of carcinogenic polycyclic aromatic hydrocarbons (PAHs) [208,209,210]. Most worryingly, the intake of a pro-inflammatory diet can exacerbate the tumourigenic effects of tobacco smoking and alcohol drinking on HNSCC and oesophageal carcinogenesis. Smoking and alcohol consumption may activate inflammatory response pathways, which increase oxidative stress and drive the production of reactive oxygen species (ROS) and reactive nitrogen species (RNS) at the damage site; this results in macromolecular damage which can support the initiation and progression of cancer [211,212,213]. Furthermore, such diets directly modify the microbiota of the oral cavity. Carbohydrate consumption is known to increase the population of *Lactobacilli* bacteria and cause dysbiosis linked to HNSCC. Importantly, dysbiosis in the oral cavity, pharynx or larynx may also lead to dysbiosis in one of the other sites [214,215,216].

It is important to emphasize that the Dietary Inflammatory Index (DII) is closely linked with oxidative stress. Certain dietary components such as saturated fatty acids (SFAs), trans isomers (TFAs), refined carbohydrates, lipids, and proteins from red meat and processed foods promote inflammation by stimulating the production of free radicals. A vicious cycle develops: a pro-inflammatory diet (high DII score) activates immunocompetent cells, which produce free radicals in a defensive response (high DII → oxidative stress). Conversely, excess free radicals damage cellular lipids, proteins and DNA. Lipid oxidation products act as second messengers that activate transcription factors, e.g., NF-κB, and other key signalling pathways, e.g., JAK/STAT3, ERK1/2/PI3K/Akt, and Nrf2/Keap1; these induce further production of pro-inflammatory cytokines, i.e., CRP, IL-1β, IL-6, and TNF-α (oxidative stress → stronger inflammation → higher DII) [139,140,141,142,143,144,145,146,147]. Therefore, adopting a diet with a low DII index, such as the Mediterranean diet (MD), macrobiotic diet, or plant-based (PB) diet incorporating anti-inflammatory components such as fruit and vegetables, contributes to the activation of anti-inflammatory and antioxidant mechanisms. These quench oxidising and inflammatory factors that may promote carcinogenesis and increase the risk of HNSCC tumours if present in squamous epithelial cells of the head and neck region [110,171,172,173,174,175,211,212,213].

Specifically, ROS play a key role in the development and progression of cancers originating from the squamous epithelium of the head and neck region. Oxidative stress is known to directly initiate carcinogenesis, including the initiation of cancers of the upper respiratory and digestive tracts [217,218,219]. Generally speaking, the development of cancerous lesions can be divided into three stages. Firstly, the initiation stage: High production of ROS factors, i.e., hydroxyl radicals, damages DNA structure, specifically targeting the nitrogen-containing guanine. These transformations result in the formation of toxic compounds such as 8-hydroxy-2′-deoxyguanosine (8-OHdG), which ultimately leads to irreversible genetic mutations and the activation of oncogenes that promote cancer development [219,220,221]. This is followed by promotion: Oxidative stress promotes proliferation and increases the number of mutant cells by influencing the activation of transcription factors such as NF-κB, p53, and Nrf2, which simultaneously promote cell division and inhibit tumour cell apoptosis [219,220,221,222,223]. Finally, the tumour undergoes progression: Cancer cells alter the local tumour microenvironment by increasing ROS production. This overproduction stimulates matrix metalloproteinases (MMPs), which destroy surrounding tissue structures, promoting cancer invasion and causing increased formation and growth of new blood vessels, which induces distant tumour formation.

Importantly, UADT tumours are particularly susceptible to ROS/RNS generation as they are directly exposed to strong oxidants from the external environment, such as tobacco smoke and alcohol, as well as poor oral hygiene. These are typical risk factors for HNCs and promote the action of toxic free radicals, weakening local host immunity. Indeed, significant increases in malondialdehyde (MDA), a product of lipid peroxidation that damages cell membranes, have been noted in the saliva of patients. The tumours are also characterised by significant depletion of antioxidant enzymes such as superoxide dismutase (SOD) and catalase. Therefore, saliva testing for pro-inflammatory factors and ROS/RNS is a promising, non-invasive method for early detection of HNSCC metastasis [219,224,225,226,227,228].

Numerous studies indicate that, like the DII/E-DII index, dietary index or pattern analysis provides a good indicator of the relationship between chronic diseases, including HNSCC, and diet [29,30,31,32,33,34,35]. For example, Saraiya et al. [34] report a consistent association between a high HEI-2005, MDS and MDS-HNC score and the development of HNSCC (ORs: 1.35, 1.13 and 1.17, respectively); this research was conducted as part of the International Head and Neck Cancer Epidemiology (INHANCE) consortium. In contrast, following a Mediterranean diet or consuming its anti-inflammatory components was associated with a significantly lower risk of HNC (OR for MDS index: 0.61 and OR for an increase of 1 unit: 0.71) [32]. Therefore, DII/E-DII and other dietary indices may have significant epidemiological significance in predicting carcinogenesis, including HNCs, and the development of other chronic lifestyle diseases. Hence, it may represent an important method for clinically examining the relationship between diet-related inflammation and the risk of HNSCC. It is important to emphasize that the analysis of DII/E-DII parameters and overall dietary patterns is already an important method in epidemiological studies; it can provide a comprehensive, reliable, and realistic picture of the relationship between diet and the risk of chronic diseases. Notably, in recent years, researchers have evaluated the holistic assessment of diet, for example, in conjunction with a healthy lifestyle, such as recreational sports, rather than formulating recommendations for individual foods and nutrients. For example, the American Cancer Society (ACS) Guidelines on Nutrition and Physical Activity for Cancer Prevention emphasise healthy food choices and physical activity when offering recommendations for individuals to reduce the risk of cancer [229,230].

As expected, the overwhelming majority of original observational studies, case-control studies, and meta-analyses indicate that a pro-inflammatory diet significantly increases the risk of developing various site-specific HNCs, i.e., oral, oropharyngeal, nasopharyngeal, hypopharyngeal, laryngeal, and oesophageal cancer [47,53,54,59,98,99,198,199,200,201,202,203,204,205,206]. However, of all the original publications, three did not demonstrate any association between dietary food groups decisive for the results of DII/E-DII and HNSCC risk, specifically laryngeal and oesophageal cancers [55,57].

### 4.1. Study Strengths and Limitations

This narrative review has several strengths. Most importantly, it was rigorously conducted and included a comprehensive review of the literature from the past decade. The articles were also analysed in accordance with the PRISMA 2020 guidelines, and the corpus included the key meta-analyses on the topic [197]. This review is the most recent to examine the observed relationship between summary DII and E-DII data and the risk of HNSCC in the global population from 2024, i.e., large cohorts of patients. To allow a clear and accurate assessment of dietary information, data on either the calculated DII or E-DII indexes in each study were used to improve comparability. The results were analysed in a group of histologically homogeneous upper aerodigestive cancers, i.e., squamous cell carcinomas (SCC). In addition, to allow a comprehensive assessment of the diversity of food consumed by patients, our analysis did not include studies examining the effects of individual products/diet components or food groups. All analysed studies used publicly available validated food frequency questionnaires (FFQs) using the international food composition table; they employed self-report questionnaires to retrospectively assess food intake, which avoided the risk of recall bias. The analysis accounted for potential confounding variables, such as age, sex, tobacco and alcohol use, socioeconomic status (SES), oral and dental health, and adjusted risk estimates, if such data were included in the original study.

However, the study has some limitations. Firstly, the final data analysis included observational studies, albeit all of the same type (case-control studies), which are subject to unavoidable bias regarding subject selection and food exposure. Importantly, when analysing the results linking high dietary inflammation index (DII) to the risk of head and neck cancer, it is essential to recognize that most data have been obtained from observational or case-control studies. While suggesting an association between the parameters studied, these do not allow for the establishment of a causal relationship.

Secondly, while HNC constitutes a group of neoplastic diseases derived from squamous epithelium, they have a complex and diverse biology; they consequently demonstrate differing responses to anti- and pro-inflammatory products, and variable activation or inhibition of signalling pathways important for carcinogenesis. Furthermore, different HNC site-subtypes may also receive differing exposures to nutritional components due to their location: the oral area and pharynx are directly exposed to dietary intake, which could yield greater negative effects than in the case of laryngeal cancer. The oesophagus also undergoes only brief exposure to dietary components before they reach the mucosa of the stomach and small intestine; this may account for the weaker effect of pro-inflammatory diets on this type of cancer. However, most researchers agree on the influence of consuming food at extremely high temperatures, which may lead to thermal injuries, ulceration, hyperplasia, and dysplasia; these can ultimately result in carcinogenesis in the oral cavity, pharynx, and oesophagus [231]. It is also possible that smoking status and alcohol consumption may modify the association between dietary intake and HNSCC [55,57,98,99,198]. However, as the numbers of individuals in the strata are relatively low in cited articles, the evidence regarding these interactions remains unclear. Moreover, alcohol, one of the risk factors for oesophageal cancer in the DII scoring system, was indicated as an anti-inflammatory compound, which may also have contributed to the heterogeneity in the results regarding HNSCC [56].

Thirdly, the meta-analyses fail to account for the diverse diets and food patterns consumed in different regions, which may complicate data analysis and prevent conclusive results. Studies have also shown a stronger association between E-DII scores and HNSCC risk in the American population than in European and Asian populations. This is to be expected as the US population is more likely to consume a Western diet, i.e., pro-inflammatory, whereas Europe and Asia tend to consume more vegetables and anti-inflammatory components such as ginger, turmeric, saffron, and thyme, and less red meat. It is also worth noting that the studies included in the meta-analyses were performed in different years and were of varying quality, which could result in discrepancies in the final data. In particular, one study included in five meta-analyses, Abe et al. [55], obtained a low NOS score (NOS score: 6), and might be a source of heterogeneity. Nevertheless, the remaining original publications demonstrated high study quality (NOS score: 7–9). Furthermore, many of the studies used different numbers of DII/E-DII components as part of data collection, i.e., from 19 to 36, and the tree research included in the meta-analyses was based on a comparatively small sample size [99,200,204].

Another important consideration is the synergistic or counteracting effects demonstrated by combinations of dietary constituents; for example, combining the high-fat diet with the Mediterranean diet may have simultaneous deleterious and protective effects, which may influence the final DII score. Specific combinations of diverse dietary components may also have complex effects on chronic inflammation and cancer [200].

Furthermore, the included studies may be characterised by heterogeneous patient samples, insufficient sample sizes from patient and control groups, and variable observation periods, which may be sources of error and potential bias. The included studies also exhibit inconsistencies in terms of the length of the selected FFQ and sample collection. Although all publications obtained dietary information through FFQs, the specific dietary components used in the studies varied in quantity, i.e., from 47 to 159 items used. Furthermore, it is important to note that different FFQs are specific to geographic regions, which may have specific foods and dietary patterns; as such, it is difficult to establish a specific global, reproducible dietary pattern that could be transferred to different populations. The selection criteria of the control group also varied between studies, and the choice of a hospital-based control or a population-based control will affect the final results, as they will have different diets.

Additionally, due to the cross-sectional design, it was not possible to determine whether the effect of DII on HNSCC incidence changes over time after implementing healthy dietary recommendations. Finally, it should be emphasized that although the DII index mathematically describes the association between multiple food components and inflammatory response (i.e., pro- and anti-inflammatory cytokine production), food components are not consumed in isolation. Therefore, DII assessments are better suited to analysing specific dietary patterns than for clinical applications. Even so, the DII index may have significant epidemiological significance in predicting chronic lifestyle diseases such as cancer, including head and neck cancer (HNC), and may be an important tool for examining the clinical relationship between diet-related inflammation and the risk of developing HNSCC.

### 4.2. Perspectives and Future Directions: Nutritional Interventions

The dietary inflammatory index (DII) can be a valuable source of information for dietitians, medical specialists, and oncologists. It can also represent a practical parameter in clinical nutrition, facilitating appropriate nutritional decisions and monitoring patients with persistent conditions associated with inflammation, such as metabolic diseases, obesity, cardiovascular and neurodegenerative disorders and diabetes, as well as human cancers of various origins. Hence, it has a number of future clinical applications. First, it enables the introduction of personalized nutrition for high-risk cancer patients, i.e., specific dietary recommendations to reduce the incidence of specific cancer types, and patients with other lifestyle diseases. It can also be of value when working with healthy individuals as prophylaxis to prevent chronic inflammation: a high DII score could identify individuals at high risk of developing neoplastic disease, and who could benefit from early dietary plans and nutritional interventions. Implementation of the DII would also allow ongoing monitoring of the resulting dietary recommendations, thus reducing the risk of cancer development and the need for interdisciplinary care of healthy individuals (preventive procedures) and patients (therapeutic treatment). It should also be noted that World Research Fund (WCRF) and the American Institute for Cancer Research (AIRC) recommendations prioritise dietary behaviour, alongside body fatness and physical activity, in cancer prevention. Studies have clearly shown that greater adherence to WCRF/AICR recommendations is associated with a reduced risk of HNSCC cancer, and can be used as a dietary intervention for cancer prevention [232,233].

Hence, DII analysis may be of value in regional and global health initiatives aimed at popularizing healthy eating and promoting equal access to healthy food, in which it could be used to identify populations at most risk of a given inflammation-related lifestyle disease. To develop healthy dietary options for individual regions of the world, with specific access to health products and regional traditions, it is also important to support reforms in the food industry, i.e., by providing easier access to minimally processed, anti-inflammatory foods, which are available at the lowest possible prices. A global problem in almost all countries is the adoption of a “modern”, yet unhealthy diet associated with a significantly higher consumption of ultra-processed foods (UPF) rich in sugars, fats, artificial flavours, preservatives and additives. Importantly, such a diet is characterized by a high pro-inflammatory potential and significantly higher DII score. Global efforts to reduce the consumption of ultra-processed foods, while promoting a nutritious anti-inflammatory diet and instilling healthy, protective dietary habits, are becoming key to effectively preventing inflammation and reducing the incidence of lifestyle diseases.

In addition, the DII index may provide insight into the impact of pro-inflammatory diets on the expression of specific genes and epigenetic modifications, and on the activity of various subpopulations of immunocompetent inflammatory cells. Genetic, epigenetic, immunological and nutrigenomic studies may be used to identify groups at particular risk of inflammatory-related diseases, as well as new potential therapeutic targets. The implementation of such findings can be supported by real-time monitoring of dietary recommendations made possible by modern technology and mobile applications; the data can be used to steer appropriate dietary choices and encourage individuals to make informed dietary decisions. As the potential of dietary inflammatory analysis becomes better understood, the integration of specific and effective dietary changes may become an indispensable tool with a real impact on dietary and therapeutic decision-making. Such integration would greatly improve health outcomes in given populations by deepening our knowledge of the complex relationships between diet and inflammation. With the completion of larger prospective studies employing validated and reliable dietary guidelines, it is possible that other clinically useful tools will be developed that will drive more in-depth research describing the relationship between diet and lifestyle diseases such as cancer. Finally, it is important to highlight the need to research the diagnostic role of the DII index or other dietary tools in relation to HPV-related head and neck cancers, whose incidence is increasing in young patients. There is also a need for further field intervention studies to provide stronger evidence for the relationship between DII and HNSCC.

The measurement of DII/E-DII indices may have value in prospective cohort studies for establishing cause-and-effect relationships between risk factors, such as diet, and the development of specific lifestyle diseases, including upper aerodigestive tract cancers (UADT). Such observational analytical studies, which track a cohort in real time from the moment of exposure until the occurrence of a specific health effect, may be used to identify specific nutrients that exacerbate or prevent UADT. Furthermore, eliminating the problem of memory bias is also important, as data is collected continuously rather than retrospectively; this can also allow the assessment of multiple regulatory mechanisms and health effects of a single dietary factor or entire groups of several nutrients.

DII/E-DII index determinations can also be used in dietary interventions. Identifying dietary components and dietary patterns with anti- and pro-inflammatory effects can support the design of diets that can improve health and support the treatment or prevention of diseases such as head and neck cancer (HNC). For example, an intervention may favour the use of the anti-inflammatory Mediterranean diet (MD), macrobiotic diet or plant-based (PB) diets (Higher DII index), while avoiding the pro-inflammatory Western Diet (WD), High-Fat Diet (HFD), and fast-food diets (Lower DII index).

Most importantly, compounds such as ω-3 fatty acids and dietary fibre, or substances such as polyphenols, play significant roles at every stage of carcinogenesis. Polyunsaturated fatty acids such as ω-3 fatty acids, particularly eicosapentaenoic acid (EPA) and docosahexaenoic acid (DHA), exhibit anti-inflammatory and anti-catabolic effects by modulating the mechanistic target of rapamycin complex 1 (mTORC1, mTORC1) and nuclear factor kappa B (NF-κB) pathways and limiting the production of proinflammatory cytokines. The current guidelines of the European Society for Clinical Nutrition and Metabolism (ESPEN) and the European Society for Medical Oncology (ESMO) recommend the consumption of EPA and DHA at a dose of ~2 g per day, preferably as part of comprehensive nutritional support.

Numerous studies indicate that including ω-3 fatty acids in the diet can support the maintenance of muscle mass, improve nutritional status, reduce inflammatory markers, and mitigate the side effects of cancer therapy. In addition, ω-3 fatty acids may support the regenerative process, reduce the risk of therapeutic complications, and improve quality of life. Moreover, studies indicate that higher blood levels of ω-3 fatty acids are associated with a significantly lower risk of cancer. Firstly, these acids are believed to support cancer prevention by inhibiting the proliferation of cancer cells: studies have shown that ω-3 fatty acids help destroy malignant and pre-malignant oral squamous cell carcinoma cells without damaging healthy tissue. Secondly, they are thought to act by reducing the proliferation of cancer cells and inhibiting angiogenesis by blocking certain oncogenes and inflammation. Thirdly, they have been found to alleviate the effects of chemotherapy: supplementation with EPA and DHA acids significantly reduces pain and the intensity of oral mucositis in patients undergoing toxic treatment. In addition, the acids offer nutritional support, which helps maintain body weight and muscle mass, and improves appetite in cancer patients undergoing chemotherapy and radiotherapy. The main sources of ω-3 fatty acids are fatty saltwater fish (wild salmon, mackerel, herring, and sardines), although they are also found in vegetable oils and nuts, including flaxseeds, chia seeds, and walnuts (a source of ALA). They can also be consumed as supplements. Most Oncology studies are based on high doses of EPA and DHA, ranging from 600 mg to 3.6 g per day [234,235,236,237].

Polyphenols exhibit potent chemopreventive and supportive effects against HNSCC, inhibiting the development of cancer cells at the stages of initiation, promotion, and progression. These natural plant compounds destroy cancer cells, primarily squamous cell carcinoma (OSCC), by inducing apoptosis, i.e., programmed cell death, and blocking their ability to migrate and metastasize. A number of polyphenols have been studied in the context of HNSCC. Epigallocatechin (EGCG), which is derived from green tea and inhibits tumour vascularization (angiogenesis), has been found to reduce the activity of EGFR receptors responsible for cell division. Resveratrol, present in grapes and berries, has been found to significantly reduce cancer cell viability and inhibit tumour growth in preclinical models. Curcumin, a component of turmeric, induces cell cycle arrest by blocking signalling pathways (e.g., Hedgehog/GLI, HH/GLI, and NF- κB). Black tea polyphenols (PBPs) have been preclinically proven to reduce the volume and number of cancerous lesions, including those in the oral cavity [238,239,240,241].

However, despite promising results, polyphenols demonstrate low solubility and poor bioavailability in the human body. Because these compounds are rapidly metabolized, advanced delivery systems (e.g., nanoparticle carriers or oral transmembrane delivery) are under development to increase their concentration directly at the tumour site. Also, large doses of purified polyphenols, as found in supplement form, can be toxic or interfere with iron absorption. Instead, experts recommend a balanced diet rich in natural sources of polyphenols [242,243,244].

Preclinical studies indicate that polyphenols affect HNSCC cancer cells, especially OSCC, in multiple ways. They have been found to induce apoptosis, i.e., activating programmed cancer cell death by inter alia regulating the p53 protein and the proapoptotic protein Bax. They also inhibit proliferation and cell cycle progression by blocking cell division and limiting the expression of growth-stimulating receptors such as EGFR. Polyphenols also exhibit anti-metastatic effects by reversing the epithelial-to-mesenchymal transition (EMT) and reducing the activity of enzymes (e.g., MMP-2) that enable cancer cells to infiltrate and migrate into tissues. They also reduce angiogenesis and hypoxia; studies have found treatment with polyphenols to reduce the secretion of VEGF, a key agent in blood vessel formation, and the levels of pro-inflammatory cytokines (such as IL-1β, IL-6, IL-8, and COX-2) that promote disease progression.

A high intake of dietary fiber is strongly linked to a significantly lower risk of developing HNSCC. Large-scale epidemiological studies, including pooled analyses from the International Head and Neck Cancer Epidemiology (INHANCE) Consortium, indicate that consuming high levels of dietary fibre is associated with up to 50% reduced risk of oral and pharyngeal cancers compared to low intake. Furthermore, emerging data indicate that high pre-treatment fiber intake may also significantly improve survival outcomes and lower all-cause mortality in patients already diagnosed with HNC [245,246,247].

Dietary fiber acts through multiple pathways to block or mitigate HNSCC carcinogenesis: (1) High-fiber diets reduce systemic and localized inflammation, lowering the risk of tissue damage that leads to malignant transformation. (2) Fiber-rich food sources are naturally dense in vitamins (such as Vitamin C and folate) and phytochemicals that neutralize free radicals and prevent DNA mutations. (3) Soluble fiber ferments in the gut, fostering beneficial microbiota that strengthen the overall immune response against cancerous cell growth. (4) Fiber promotes satiety and helps maintain a healthy weight, indirectly lowering the risk of obesity-related malignancies. The greatest benefits are derived from unrefined foods rich in legumes (beans and lentils deliver both protective fiber and cell-repairing folate), fresh fruits (apples, pears, oranges, and bananas provide a combination of soluble fiber and crucial antioxidants), and whole grains (oats, brown rice, and whole-wheat cereals provide dense grain fibers) [248,249].

Such clinical management is a key element of evidence-based medicine (EBM), integrating knowledge of anti- and pro-inflammatory mechanisms with daily eating habits. Moreover, nutritional interventions can prevent the consequences of cancer itself, such as malnutrition and cachexia; it can also increase tolerance of oncological treatment and improve quality of life. Replacing nutritional deficiencies resulting from poor nutritional status can often prevent surgery or the administration of a full dose of chemotherapy. The European Society for Clinical Nutrition and Metabolism (ESPEN) recommends nutritional support as an integral part of therapy from diagnosis until the completion of oncological treatment. Nutritional interventions, depending on the overall health of a cancer patient, their nutritional status, and the functioning of the gastrointestinal tract, involve various forms of support, such as dietary counselling, oral nutritional supplements, and enteral and parenteral nutrition.

Selecting a daily diet or using commercially prepared preparations based on calculated DII/E-DII indices could be a potential clinical target leading to the induction of protective anti-inflammatory dietary mechanisms. The role of dietary counselling is to familiarize cancer patients with the potential for valuable modifications to a traditional, often unhealthy or nutrient-limited, home-cooked diet, including by enriching meals with natural sources of energy and protein. Such a balanced diet helps offset the increased metabolism associated with cancer and protects against weight loss, while also facilitating tissue regeneration, wound healing, and muscle preservation. Oral nutritional supplements (ONS), characterized by high protein and calorie values, can be used as supplements. Finally, enteral nutrition is crucial. This involves administering nutritional mixtures directly to the stomach or small intestine through a tube (probe) or gastrostomy (e.g., PEG). Parenteral nutrition, another form of food, involves the intravenous administration of balanced and appropriately selected nutrients. It is used when the digestive tract is not functioning properly (e.g., the frequent problem of oesophageal strictures in patients with HNSCC), thus protecting the body against deficiencies of valuable dietary components and enhancing the diet’s anti-inflammatory effects.

In the case of head and neck cancers, stratified analyses based on HPV status will also be of potential importance. Answering the question of whether specific nutritional factors or dietary patterns, reflected in the DII/E-DII score, may indicate or be associated with the risk of HPV infection will allow for the elimination of food components from the daily diet that increase the risk of developing HPV-related HNSCC, typical of young people. Unfortunately, it is impossible to find publications that would directly refer to and describe the relationship, including the cause-and-effect relationship, between specific dietary components or nutritional patterns and HPV infection in HPV-positive cancers.

Finally, an interesting and necessary supplement would be potential research examining the relationship between long-term changes in the DII/E-DII index and the prognosis of lifestyle diseases, including head and neck cancer. Such observations would allow for the avoidance or elimination of negative dietary factors and nutrients that promote the inflammatory process leading to the development of these diseases. Proper monitoring of DII/E-DII indexes in patients over a long period of follow-up would allow for prediction of the patients’ future outcomes, thus enabling dietary adjustments as a prognostic measure. Unfortunately, this problem also remains undescribed in the literature and requires further observational studies, case-control studies, and prospective studies.

Regardless of the above-mentioned limitations, it should be emphasized that DII/E-DII has a great epidemiological significance in predicting chronic disease, including cancer. It may also be a valid method for estimating the relationship between diet-induced inflammation and the risk of neoplastic disease, such as the heterogeneous group of head and neck cancers.

## 5. Conclusions

More pro-inflammatory diets and food types, indicated by a higher dietary inflammatory index (DII) or energy-adjusted dietary inflammatory index (E-DII), are associated with a greater risk of developing head and neck squamous cell carcinomas (HNSCCs). Such diets tend to be rich in processed foods with high levels of refined grains, simple sugars, red and processed meat, eggs, and high-fat dairy. Conversely, the consumption of a diet with anti-inflammatory properties, and hence a lower DII score, may protect against the initiation and development of HNSCC by reducing persistent inflammation in the body. Such healthy dietary patterns are typically based on the consumption of unprocessed foods, with plenty of fruits and vegetables, oily fish and healthy fats, as well as whole grain products containing large amounts of antioxidants, polyunsaturated fatty acids (PUFAs), fibre and natural spices. Such diets may also contain large amounts of vitamins and certain trace elements.

Our findings indicate that dietary inflammatory index (DII) is a valuable and clinically necessary tool for preventing various lifestyle diseases associated with inflammation and predicting their development. Its use represents a new direction for improving public health when used in combination with genetic, epigenetic, immunological, and nutrigenomic data. Further research should include the implementation of a broader population database that accounts for cultural and dietary differences unique to geographical areas. It should also incorporate an expanded range of readily available anti-inflammatory functional products. There is also a need to harmonize dietary guidelines based on emerging evidence. Research into DII should also emphasise personalized nutrition, aimed at inter alia preventing the risk of cancer, the second leading cause of death globally. In any case, additional research should incorporate consistent methodologies aimed at validating these tools and better understanding the mechanisms and the relationship between diet, neoplastic diseases, and other lifestyle diseases.

## Figures and Tables

**Table 1:** Search strategy.

Database	Search Strategy	Records
PubMed	(“Head and neck neoplasm”[All Fields] OR “HNSCC”[All Fields] OR “Squamous cell carcinoma of head and neck”[All Fields] OR “Head and neck cancer”[All Fields] OR “Head and neck carcinoma”[All Fields] OR “Upper aerodigestive tract cancers”[All Fields] OR “Upper aerodigestive tract neoplasms”[All Fields] OR “UADT”[All Fields] OR “Oesophageal cancer”[All Fields] OR “Oral cancer”[All Fields] OR “Mouth Neoplasm”[All Fields] OR “Nasopharyngeal cancer”[All Fields] OR “Oropharyngeal cancer”[All Fields] OR “Laryngeal cancer”[All Fields] OR “Nasopharyngeal neoplasm”[All Fields] OR “Oropharyngeal neoplasm”[All Fields] OR “Laryngeal neoplasm”[All Fields] AND (“dietary inflammatory index”[All Fields] OR “DII”[All Fields] OR “dietary habit”[All Fields] OR “eating pattern”[All Fields] OR “food pattern”[All Fields] OR “energy-adjusted dietary inflammatory index”[All Fields] OR “E-DII”[All Fields] OR “inflammatory dietary pattern”[All Fields] OR “dietary inflammation score”[All Fields] OR “anti- inflammatory diet”[All Fields] OR “pro-inflammatory diet”[All Fields] OR “diet”[All Fields]	628
Embase	(‘head and neck neoplasm’/exp OR ‘head and neck neoplasm’ OR ‘HNSCC’/exp OR ‘HNSCC’ OR ‘squamous cell carcinoma of head and neck’/exp OR ‘squamous cell carcinoma of head and neck’ OR ‘head and neck cancer’/exp OR ‘head and neck cancer’ OR ‘head and neck carcinoma’/exp OR’ head and neck carcinoma’ OR ‘upper aerodigestive tract cancers’/exp OR ‘upper aerodigestive tract cancers’ OR ‘upper aerodigestive tract neoplasms’/exp OR ‘upper aerodigestive tract neoplasms’ OR ‘UADT’/exp OR ‘UADT’ OR ‘oesophageal cancer’/exp OR ‘oesophageal cancer’ OR ‘oral cancer’/exp OR ‘oral cancer’ OR’ mouth neoplasm’/exp OR ‘mouth neoplasm’ OR ‘nasopharyngeal cancer’/exp OR ‘nasopharyngeal cancer’ OR ‘oropharyngeal cancer’/exp OR ‘oropharyngeal cancer’ OR ‘laryngeal cancer’/exp OR ‘laryngeal cancer’ OR ‘nasopharyngeal neoplasm’/exp OR ‘nasopharyngeal neoplasm’ OR ‘oropharyngeal neoplasm’/exp OR ‘oropharyngeal neoplasm’ OR ‘laryngeal neoplasm’/exp OR ‘laryngeal neoplasm) AND (‘dietary inflammatory index’/exp OR ‘dietary inflammatory index’ OR ‘DII’/exp OR ‘DII’ OR ‘dietary habit”/exp OR ‘dietary habit’ OR ‘eating pattern’/exp OR ‘eating pattern’ OR ‘food pattern’/exp OR ‘food pattern’ OR ‘energy-adjusted dietary inflammatory index’/exp OR ‘energy-adjusted dietary inflammatory index’ OR ‘E-DII’/exp OR ‘E-DII’ OR ‘inflammatory dietary pattern’/exp OR ‘inflammatory dietary pattern’ OR ‘dietary inflammation score”/exp OR ‘dietary inflammation score’ OR ‘anti- inflammatory diet”/exp OR ‘anti- inflammatory diet’ OR ‘pro-inflammatory diet/exp’ OR ‘pro-inflammatory diet’ OR ‘diet/exp OR ‘diet’)	47
Web of science	ALL = ((“Head and neck neoplasm” OR “HNSCC” OR “Squamous cell carcinoma of head and neck” OR “Head and neck cancer” OR “Head and neck carcinoma” OR “Upper aerodigestive tract cancers” OR “Upper aerodigestive tract neoplasms” OR “UADT” OR “Oesophageal cancer” OR “Oral cancer” OR “Mouth Neoplasm” OR “Nasopharyngeal cancer” OR “Oropharyngeal cancer” OR “Laryngeal cancer” OR “Nasopharyngeal neoplasm” OR “Oropharyngeal neoplasm” OR “Laryngeal neoplasm”) AND (“dietary inflammatory index” OR “DII” OR “dietary habit” OR “eating pattern” OR “food pattern” OR “energy-adjusted dietary inflammatory index” OR “E-DII” OR “inflammatory dietary pattern” OR “dietary inflammation score” OR “anti- inflammatory diet” OR “pro-inflammatory diet” OR “diet”))	423

**Table 2:** The characteristics of observational (case-control) studies calculating HNSCC risk in relation to DII/E-DII score.

Author/ Publication Year	Case-Controls Studies			
	Region	Characteristics	Study Design	Results
Narmcheshm et al. [57]/2024	Iran	Cases/controls	879 cases/3409 controls	The risk of all HNC with categorical DII (tertiles T1–T3): - the highest DII tertile associated with increased risk of all HNC: OR_T3vs.T1_ was 1.31 (95% CI: 1.06–1.62, *p*_trend_ = 0.013) The risk of LOSCC with categorical DII: - significant association noted between LOSCC and DII: the pooled OR_T3vs.T1_ was 1.56 (95% CI: 1.16–1.66; *p*_trend_ = 0.004) The risk of PSCC with categorical DII: - significant association observed between PSCC and DII: the pooled OR_T3vs.T1_ was 2.08 (95% CI: 1.14–3.79; *p*_trend_ = 0.02) The risk of LSCC with categorical DII: - no significant association observed between DII and laryngeal cancer Subgroup analysis: - significant interaction between cases who used tobacco with higher DII score on the risk of HNC compared to participants who did not use tobacco and were in T1 of DII: the pooled OR_T3vs.T1_ was 2.52 (95% CI: 1.78–3.57; p*_interaction_* = 0.03) - cases who did not use tobacco or opium and were in T3 of DII had greater risk of HNC: the pooled OR_T3vs.T1_ was 1.66 (95% CI: 1.20–2.29; p*_interaction_* = 0.03)
		Period (years)	2018–2020	
		Cancer site	Oesophageal cancer (ESCC), lip and oral cancer (LOSCC), pharyngeal (PSCC), laryngeal (LSSC)	
		FFQ (items)/ DII/E-DII components	130-item FFQ 38 E-DII components	
		NOS score	NR	
		Adjustments	Age, sex, race, tobacco use, alcohol use, income, education,	
Bao et al. [198]/2020	China	Cases/controls	295 cases/425 controls	The risk of OSCC with categorical E-DII (quartile Q1–Q4): - the highest E-DII quartile was associated with an increased risk of OSCC: the pooled OR_Q4vs.Q1_ was 2.56 (95% CI: 1.67 –3.93, p*_trend_* < 0.001) - after adjusting for covariates, the highest E-DII quartile was associated with an increased risk of OSCC: an adjusted OR_Q4vs.Q1_ was 2.57 (95% CI: 1.54, 4.29, p*_trend_* = 0.013) Dose-response association between E-DII and OSCC: - OR for a 1-unit increment in the E-DII was 1.04 (95% CI: 1.01–1.06) - the OR for a 1-unit increment in the E-DII after adjusting for selected covariates was 1.03 (95% CI: 1.00–1.06) Subgroup analysis: - significant interaction observed between E-DII and oral hygiene for oral cancer: the pooled OR_Q4vs.Q1_ values were 1.96 (95% CI: 0.96–4.00) for good hygiene and 4.23 (95% CI: 1.83–9.81) for poor hygiene, p*_interaction_* < 0.001 - stronger association noted between E-DII and OSCC among male subjects: the pooled OR_Q4vs.Q1_ was 2.94 (95% CI: 1.44–6.00); >60 yrs. OR_Q4vs.Q1_ was 3.13 (95% CI: 1.19–8.27); never-smoking subjects OR_Q4vs.Q1_ was 2.47 (95% CI: 1.25–4.86); alcohol drinkers OR_Q4vs.Q1_ was 4.35 (95% CI: 1.54–12.25); subjects with poor hygiene (OR_Q4vs.Q1_ was 4.23 (95% CI: 1.83–9.81) - No statistically significant differences were observed between the highest E-DII quartile vs. the lowest E-DII quartile on sex, age, occupation, residence and BMI (all *p* > 0.05)
		Period (years)	2010–2018	
		Cancer site	Oral cancer (OSCC)	
		FFQ (items)/ DII/E-DII components	127-item FFQ 25 E-DII components	
		NOS score	7	
		Adjustments	Age, sex, education level, BMI, tobacco smoking, alcohol drinking, occupation	
Secchi et al. [99]/2019	Argentina	Cases/controls	27 cases/86 controls	The risk of OSCC with categorical E-DII (tertiles T1-T3): - the highest E-DII tertile was associated with an increased risk of OSCC: the pooled OR_T3vs.T1_ was 18.46 (95% CI: 2.28 –149.72, p*_trend_* = 0.003) after adjusting for covariates such as alcohol drinking and tobacco smoking - a significant association was noted between E-DII (continuous) and OSCC: OR was 1.69 (95% CI: 1.18–2.43, p*_trend_* < 0.0029)
		Period (years)	2012–2015	
		Cancer site	Oral cancer (OSCC)	
		FFQ (items)/ DII/E-DII components	159-item FFQ 22 E-DII components	
		NOS score	7	
		Adjustments	Age, sex, occupation, education level, residence, BMI, family history of cancer, tobacco smoking, alcohol drinking, tea consumption, oral hygiene score	
Mazul et al. [98]/2018 (Carolina Head and Neck Cancer Epidemiology—CHANCE study)	American	Cases/controls	1389 cases/1396 controls	The risk of all HNC with categorical DII (quartiles Q1–Q4): - the highest E-DII quartile was associated with an increased risk of HNSCC: the pooled OR_Q4vs.Q1_ was 2.91 (95% CI: 2.16 –3.95, p*_trend_* < 0.001) The risk of LSCC with categorical DII: - the highest E-DII quartile was associated with increased risk of OSCC: the pooled OR_Q4vs.Q1_ was 2.47 (95% CI: 1.34–4.75, p*_trend_* = 0.01) The risk of OPSCC with categorical DII: - the highest E-DII quartile was associated with increased risk of OPSCC: the pooled OR_Q4vs.Q1_ was 2.92 (95% CI: 1.86–4.70, p*_trend_* < 0.001) Subgroup analysis: - significant interaction between cases who used tobacco and a higher additive interaction with E-DII: RERI was 2.83 (95% CI: 1.36–4.30) - significant interaction between cases who used alcohol and a higher additive interaction with E-DII: RERI was 1.75 (95% CI: 0.77–2.75)
		Period (years)	2002–2006	
		Cancer site	All head and neck cancer (HNSCC), oral cancer (OSCC), oropharyngeal (OPSCC), hypopharyngeal (HPSCC), laryngeal (LSSC)	
		FFQ (items)/ DII/E-DII components	72-item FFQ 27 E-DII components	
		NOS score	7	
		Adjustments	Age, sex, education level, BMI, tobacco smoking, total lifetime alcohol drinking, occupation, cancer site	
Abe et al. [55]/2018	Japan	Cases/controls	1028 cases/3081 controls	The risk of UADT with categorical DII (quartile Q1-Q4): - highest DII quartile was associated with an increased risk of UADT: the pooled OR_Q4vs.Q1_ was 1.96 (95% CI: 1.58–2.43, p*_trend_* < 0.001) - after adjusting for covariates, the highest DII quartile was associated with an increased risk of UADT: adjusted OR_Q4vs.Q1_ was 1.73 (95% CI: 1.37–2.20, p*_trend_* < 0.001) The risk of HNSCC with categorical DII (quartile Q1-Q4): - the highest DII quartile was associated with increased risk of HNSCC: the pooled OR_Q4vs.Q1_ was 2.16 (95% CI: 1.63–2.87, p*_trend_* < 0.001) - after adjusting for covariates, highest DII quartile was associated with increased risk of HNSCC: adjusted OR_Q4vs.Q1_ was 1.92 (95% CI: 1.42–2.59, p*_trend_* < 0.001) The risk of ESCC with categorical DII (quartile Q1–Q4): - the highest DII quartile was associated with increased risk of ESCC: the pooled OR_Q4vs.Q1_ was 1.72 (95% CI: 1.24–2.38, p*_trend_* = 0.03) - after adjusting for covariates, highest DII quartile was associated with increased risk of ESCC: an adjusted OR_Q4vs.Q1_ was 1.71 (95% CI: 1.54–1.90, p*_trend_* = 0.07) The risk of subsites of HNSCC with categorical DII (quartile Q1–Q4): - OSCC: the pooled OR was OR was 2.42 (95% CI: 1.58–3.71, p*_trend_* < 0.001) and 2.38 (95% CI: 1.52–3.72, p*_trend_* < 0.001) after adjusting for covariate OR - NSCC: the pooled OR was 3.65 (95% CI: 1.10–12.11, p*_trend_* = 0.05) and 4.99 (95% CI: 1.14–21.79, p*_trend_* = 0.09) after adjusting for covariate OR - OPSCC: the pooled OR was 1.63 (95% CI: 0.72–7.97, p*_trend_* = 0.21) and 1.71 (95% CI: 0.65–4.50, p*_trend_* = 0.27) after adjusting for covariate OR - HPSCC: the pooled OR was 3.03 (95% CI: 1.15–7.97, p*_trend_* = 0.07) and 4.05 (95% CI: 1.24–13.25, p*_trend_* = 0.04) after adjusting for covariate OR Risk of HPSCC and NPSCC were greatly elevated. No significant association was observed between DII and risk of either LSCC or OPSCC (*p* > 0.05) Subgroup analysis: - significant interaction noted between cases who used tobacco and a higher additive interaction with DII: OR was 1.48 (95% CI: 1.10–1.78, p*_trend_* = 0.007) - significant interaction observed between cases who used alcohol and a higher additive interaction with DII: OR was 1.59 (95% CI: 1.20–2.12, p*_trend_* = 0.0073)
		Period (years)	2001–2005	
		Cancer site	Upper aerodigestive tract cancer (UADT) such as oral cancer (OSCC), nasopharyngeal (NPSCC), oropharyngeal (OPSCC), hypopharyngeal (HPSCC), laryngeal cancer (LSCC) and oeosopheal cancer (ESCC) and all head and neck cancer (HNSCC)	
		FFQ (items)/ DII/E-DII components	47-item FFQ 19 DII components	
		NOS score	6	
		Adjustments	Age, sex, occupation group, tobacco smoking, alcohol drinking, flushing phenotype, teeth (amount)	
Tang et al. [199]/2018	Chinese	Cases/controls	395 cases/380 controls	The risk of ESCC with categorical DII (quartile Q1–Q4): - the highest DII quartile was associated with an increased risk of ESCC: the pooled OR_Q4vs.Q1_ was 2.55 (95% CI: 1.61–4.06, p*_trend_* < 0.001) Subgroup analysis: - significant interaction noted between tobacco smokers and a higher additive interaction with E-DII: the pooled OR was 1.59 (95% CI: 1.07–2.36, p*_trend_* = 0.08) - significant interaction observed between ethnic minority group (i.e., not Han Chinese) and a higher additive interaction with E-DII: the pooled OR was 1.86 (95% CI: 1.24–2.80, p*_trend_* = 0.002) - significant interaction between lower education and higher additive interaction with E-DII: the pooled OR was 1.82 (95% CI: 1.09–3.06)
		Period (years)	2008–2009	
		Cancer site	Oesophageal cancer (ESCC)	
		FFQ (items)/ DII/E-DII components	137-item FFQ 22 E-DII components	
		NOS score	8	
		Adjustments	Age, sex, ethnic group, education, BMI, total energy intake, smoking status, alcohol drinking, family history of cancer	
Shivappa et al. [200] 2017	Italy	Cases/controls	946 cases/2492 controls	The risk of OSCC + PSCC with categorical DII (quartile Q1–Q4): - the highest DII quartile was associated with increased risk of OSCC + PSCC: the pooled OR_Q4vs.Q1_ was 1.80 (95% CI: 1.36–2.38, p*_trend_* < 0.0001) The risk of OSCC with categorical DII (quartile Q1–Q4): - the highest DII quartile associated with increased risk of OSCC; the pooled OR_Q4vs.Q1_ was 2.08 (95% CI: 1.47–2.93, p*_trend_* < 0.0001) The risk of PSCC with categorical DII (quartile Q1–Q4): - the highest DII quartile associated with increased risk of OPSCC: the pooled OR_Q4vs.Q1_ was 1.60 (95% CI: 0.97–2.63) - the highest DII quartile associated with increased risk of HPSCC: the pooled OR_Q4vs.Q1_ was 1.64 (95% CI: 0.93–2.89) Dose-response association between DII and OSCC + PSCC: - the pooled RR for a 1-unit increment in the DII: OR*_continues_* was 1.17 (95% CI: 1.10–1.25) Subgroup analysis: - significant association between the highest DII and case age; risk of OSCC + PSCC was greater among population with age ≥ 60 yrs. (the pooled OR was 2.12, 95% CI: 1.42–3.16, p*_regression_* < 0.0001) - significant association between the highest DII and sex; risk of OSCC + PSCC was greater among women (the pooled OR was 3.30, 95% CI: 1.95–5.57, p*_regression_* < 0.0001) - significant association noted between the highest DII and education; risk of OSCC + PSCC was greater among less educated subjects (the pooled OR was 1.62, 95% CI: 1.09–2.41, p*_regression_* = 0.0002) - significant association between highest DII and alcohol consumption; risk of OSCC + PSCC was greater among never drinkers (the pooled OR was 1.72, 95% CI: 1.09–2.72, p*_regression_* < 0.0001) - significant interaction between cases who used tobacco (smokers vs. never/ex-smokers) and higher additive interaction with E-DII; the pooled OR was 4.43 (95% CI: 3.40–5.78; RERI was 1.31 (*p* = 0.01) - significant interaction between alcohol consumption (never to moderate drinkers vs. heavy drinkers) and higher additive interaction with E-DII: the pooled OR was 5.87 (95% CI: 4.41–7.82; RERI was 2.43 (*p* < 0.001)
		Period (years)	1992–2009	
		Cancer site	Oral cancer (OSCC) and pharyngeal cancer (PSCC): oropharyngeal (OPSCC), hypopharyngeal (HPSCC)	
		FFQ (items)/ DII/E-DII components	78-item FFQ 15 E-DII components	
		NOS score	7	
		Adjustments	Age, sex, non-alcohol energy intake, study center, year of interview, education, body mass index, tobacco smoking, alcohol drinking	
Lu et al. 2016 [201]/2016	Sweden	Cases/controls	167 cases/820 controls	The risk of ESCC with categorical E-DII (quartile Q1–Q4): - the highest E-DII quartile was associated with an increased risk of ESCC: the pooled OR_Q4vs.Q1_ was 4.35 (95% CI: 2.24–8.43, p*_trend_* = 0.0001) Subgroup analysis: - significant interaction between higher BMI (≥25 kg/m^2^) and higher additive interaction with E-DII: the pooled OR was 6.60 (95% CI: 1.92–22.70, p*_trend_* = 0.006)
		Period (years)	1994–1997	
		Cancer site	Oesophageal cancer (ESCC)	
		FFQ (items)/ DII/E-DII components	63-items FFQ 36 E-DII components	
		NOS score	9	
		Adjustments	Age, sex, energy, education, tobacco smoking, alcohol intake, physical activity	
Shivappa et al. [202]/2016	Italy	Cases/controls	198 cases/594 controls	The risk of NPSCC with categorical E-DII (tertile T1–T3): - the highest E-DII quartile was associated with an increased risk of NPSCC when adjusted only for energy intake: an adjusted OR _T3 vs. T1_ was 1.67 (95% CI: 1.13–2.48, p*_trend_* = 0.006) After adjusting for other covariates: - the highest E-DII quartile was associated with an increased risk of NPSCC when adjusted for study centre, place of living, sex, age, year of interview and energy intake: adjusted OR_T3vs.T1_ was 1.58 (95% CI: 1.04–2.40, p*_trend_* = 0.01) - the highest E-DII quartile was associated with increased risk of NPSCC when adjusted for study centre, place of living, sex, age, year of interview education, smoking, alcohol drinking and energy intake: adjusted OR_T3vs.T1_ was 1.64 (95% CI: 1.06–2.55, p*_trend_* = 0.01) The risk of NPSCC with continuous E-DII: - higher E-DII was associated with an increased risk of NPSCC when adjusted only for energy intake: adjusted OR_T3vs.T1_ was 1.20 (95% CI: 1.07–1.34) After adjusting for other covariates: - higher E-DII was associated with increased risk of NPSCC when adjusted for study centre, place of living, sex, age, year of interview and energy intake: adjusted OR_T3vs.T1_ was 1.18 (95% CI: 1.04–1.34) - higher E-DII was associated with increased risk of NPSCC when adjusted for study centre, place of living, sex, age, year of interview education, smoking, alcohol drinking and energy intake: adjusted OR_T3vs.T1_ was 1.19 (95% CI: 1.05–1.36)
		Period (years)	1992–2008	
		Cancer site	Nasopharyngeal cancer (NPSCC)	
		FFQ (items)/ DII/E-DII components	78-item FFQ 31 E-DII components	
		NOS score	7	
		Adjustments	Study centre, place of living, sex, age, year of interview, education, tobacco smoking, alcohol drinking, energy intake	
Shivappa et al. [203]/2016	Italy	Cases/controls	460 cases/1088 controls	The risk of LSCC with categorical E-DII (quartile Q1–Q4): - the highest E-DII quartile was associated with increased risk of LSCC: the pooled OR_Q4vs.Q1_ was 3.30 (95% CI: 2.06–5.28, *p_trend_* < 0.0001) Dose-response association between E-DII and LSCC: - the pooled RR for a 1-unit increment in the E-DII was 1.27 (95% CI: 1.15–1.40, *p_linearity_* < 0.001) Subgroup and sensitivity analysis: - a significant association was noted between the highest E- DII vs. lowest E-DII level and age: the risk of LSCC was greater among population aged <60 yrs. (the pooled OR was 4.68, 95% CI: 2.26–9.67, p*_trend_* < 0.0001); with higher education (the pooled OR was 3.92, 95% CI: 1.88–8.19, p*_trend_* < 0.0001); with higher BMI (≥25 kg/m^2^) (the pooled OR was 3.62, 95% CI: 1.96–6.69, p*_trend_* = 0.0001) - significant association disclosed between the highest E-DII level and tobacco smoking; the risk of LSCC was greater for current smoker vs. never/ex-smokers (the pooled OR was 6.64, 95% CI: 4.52–9.74) - significant association observed between the highest E- DII level and alcohol consumption; risk of LSCC was greater for heavy drinkers vs. never to moderate drinkers (the pooled OR was 5.82, 95% CI: 3.62–9.37)
		Period (years)	1992–2000	
		Cancer site	Laryngeal cancer (LSCC)	
		FFQ (items)/ DII/E-DII components	78-item FFQ 31 E-DII components	
		NOS score	8	
		Adjustments	Age, sex, centre, education, BMI, tobacco smoking, alcohol consumption, non-alcohol energy intake	
Shivappa et al. [204]/2015	Iran	Cases/controls	47 cases/96 controls	The risk of ESCC with categorical E-DII (dichotomous D1–D2): - the highest DII level quartile was associated with increased risk of ESCC, the pooled OR_D2vs.D1_ was 7.55 (95% CI: 2.63–21.70, *p_trend_ =* 0.0002) after adjusting for age - the highest DII level was associated with increased risk of ESCC, the pooled OR_D2vs.D1_ was 8.24 (95% CI: 2.03–33.47, *p_trend_ =* 0.003) after adjusting for age, energy, sex, BMI, physical activity, smoking, gastro-oesophageal reflux The association between continuous E-DII and LSCC: - the pooled RR for continuous DII was 3.10 (95% CI: 1.83–5.24, *p_linearity_* < 0.001) after adjusting for age - the pooled RR for continuous DII was 3.58 (95% CI: 1.76–7.26, *p_linearity_ =* 0.0004) after adjusting for age
		Period (years)	NR	
		Cancer site	Oesophageal cancer (ESCC)	
		FFQ (items)/ DII/E-DII components	125-item FFQ 27 E-DII components	
		NOS score	7	
		Adjustments	Age, energy, sex, BMI, education, physical activity, smoking gastrooesophageal reflux	
Shivappa et al. [205]/2015	Italy	Cases/controls	304 cases/743 controls	The risk of ESCC with categorical E-DII (quintiles Q1–Q5): - the highest DII level was associated with increased risk of ESCC, conditioned OR_Q5vs.Q1_ was 3.27 (95% CI: 2.02–5.30, *p_trend_* < 0.0001) after adjusting for age, sex, year of interview and place of residence; multivariable OR was 2.47 (95% CI: 1.40–4.36, *p_trend_* < 0.0001) after adjusting for age, sex, year of interview, place of residence, education, alcohol consumption, tobacco smoking, BMI, physical activity, aspirin use and energy No association was found with sex, alcohol drinking and tobacco smoking. An association between continuous E-DII and ESCC: - the pooled RR for continues DII was 1.39 (95% CI: 1.25–1.54) after adjusting for age, sex, year of interview and place of residence - the pooled RR for continues DII was 1.23 (95% CI: 1.10–1.38) after adjusting for age, sex, year of interview, place of residence, education, alcohol drinking, tobacco smoking, BMI, physical activity, aspirin use and energy
		Period (years)	1992–2010	
		Cancer site	Oesophageal cancer (ESCC)	
		FFQ (items)/ DII/E-DII components	78-item FFQ 31 E-DII components	
		NOS score	8	
		Adjustments	Age, sex, year of interview, area of residence, education, smoking, alcohol drinking, BMI, physical activity, aspirin use	

DII: Dietary inflammatory index; E-DII, energy adjusted DII; NR: not reported; NE: not estimated; OR/HR/RR: odds ratio/hazard ratio/risk ratio; CI: confidence interval; FFQs, food frequency questionnaires; SES: socio-economic status; NOS: the Newcastle-Ottawa scale; DMTF: decayed, missing or filled teeth; RERI: smoking/alcohol relative excess risk due to interaction; LOSCC: lip and oral cavity; OSCC: oral squamous cell cancer; PSCC: pharyngeal squamous cell cancer; OPSCC: oropharyngeal squamous cell cancer; LSCC: laryngeal squamous cell carcinoma; NPSCC: nasopharyngeal squamous cell carcinoma; UADT: upper aerodigestive tract squamous cell carcinoma.

**Table 3:** The selected meta-analyses evaluating risk of cancer in relation to DII/E-DII score in HNC patients.

Author/ Publication Year	Meta-Analyses			
	Period Review (Years)	Included Authors of Observational Studies	Study Design	Results
Shrivastava et al. [59]/2024	2017–2020	Bao et al./2020 [198]	- 5 case-control studies (2621 cases/6184 controls) - oral (OSSC) and oropharyngeal squamous cell cancers (OPSCC) - 47–159-item FFQs (27–31 DII/E-DII components) - DII/E-DII of (1) the highest category vs. the lowest category - the covariates adjusted: participant recruitment location, study duration, study quality, NOS score, number of cases, number of DII components, E-DII, age, sex	The risk of OSCC + OPSCC with categorical DII: - the highest DII vs. the lowest DII level: the pooled OR was 2.28 (95% CI: 1.70–3.06), with heterogeneity (*I*^2^ = 38%, *p* = 0.17) for OSCC - the highest DII vs. lowest DII level: the pooled OR was 2.65 (95% CI: 1.76–3.99), with extremely low heterogeneity (*I*^2^ = 0%, *p* = 0.17) for OPSCC - combined OR for OSCC + OPSCC was 2.32 (95% CI: 1.84–2.94), with heterogeneity (*I*^2^ = 28%, *p* = 0.22) *p* value for overall effect was significant (*p* < 0.00001)
		Secchi et al./2019 [99]		
		Mazul et al./2018 [98]		
		Abe et al./2018 [55]		
		Shivappa et al./2017 [200]		
Luo et al. [206]/ 2022	2017–2020	Bao et al./2020 [198]	- 5 case-control studies (1278 cases/5137 controls) - oral cancer (OSSC) - 47–159-item FFQs (22–31 DII/E-DII components) - DII/E-DII of the highest category vs. the lowest category (1); higher category vs. lower category (2); and a 1-unit increment in DII/E-DII (3) - the covariates adjusted: geographic area, study duration, study quality, NOS score, number of cases, number of DII components, E-DII, age, sex, SES, BMI, and family history	The risk of OSCC with categorical DII: - the highest DII vs. the lowest DII level: the pooled OR was 2.35 (95% CI: 1.88–2.94), with low heterogeneity (*I*^2^ = 8.1%, *p* = 0.360) - higher DII vs. lower DII level: the pooled OR was 1.79 (95% CI: 1.49–2.15), with extremely low heterogeneity (*I*^2^ = 0.0%, *p* = 0.614) Dose-response association between DII and OSCC: - the pooled RR for a 1-unit increment in DII was 1.17 (95% CI: 1.05–1.30), however, significant heterogeneity was noted among the included studies (*I*^2^ = 86.1%, *p_heterogeneity_ =* 0.001) - no evidence of a nonlinear association of DII with oral cancer (*p_non-linearity_* = 0.752) - the risk of oral cancer increased linearly with increments in DII score (*p_linearity_* < 0.001) Subgroup and sensitivity analysis: - for the highest DII vs. the lowest DII and higher DII vs. lower DII: the associations between DII and oral cancer risk were consistent across strata of all the analysed factors (all *p_heterogeneity_* > 0.05), except for the highest vs. lowest category comparison for SES category (2.29 vs. 18.46, *p_heterogeneity_* = 0.052) - risk of oral cancer for a 1-unit increment in DII appeared to be more pronounced in those unadjusted subgroups by SES (1.69 vs. 1.14, *p_heterogeneity_* = 0.039) and family history of cancer (1.19 vs. 1.03, *p_heterogeneity_* < 0.001)
		Secchi et al./2019 [99]		
		Mazul et al./2018 [98]		
		Abe et al./2018 [55]		
		Shivappa et al./2017b [200]		
Zhu et al. [54]/ 2020	2015–2019	Abe et al./2018 [55]	- 8 case-control studies (2556 cases/5454 controls) - oesophageal cancer (ESCC) and upper aerodigestive tract cancer (UADT) such as oral cancer (OSCC), nasopharyngeal (NPSCC), oropharyngeal (OPSCC), hypopharyngeal (HPSCC), laryngeal cancer (LSCC) - 120–137-item FFQs (19–36 DII components) - DII/E-DII of the highest category vs. the lowest category (1); higher category vs. lower category (2); and a 1-unit increment in DII/E-DII (3) - the covariates adjusted: cancer site, ethnicity, age, length of follow-up, study quality, DII components, E-DII score	The risk of ESCC with categorical DII: - the highest DII vs. the lowest DII: the pooled OR was 2.29 (95% CI: 1.75–3.00), with heterogeneity (*I*^2^ = 61.1%, *p* = 0.025) - higher DII vs. lower DII: the pooled OR was 1.61 (95% CI: 1.40–1.87), with heterogeneity (*I*^2^ = 45.7%, *p* = 0.087) Dose-response association between DII and ESCC: - the pooled RR for a 1-unit increment in the DII was 1.18 (95% CI: 1.13–1.24) with heterogeneity (*I*^2^ = 51.4%, *p* = 0.055) The risk of UADT + ESCC with categorical DII: - the highest DII vs. the lowest DII: the pooled OR was 2.07 (95% CI: 1.82–2.35), with heterogeneity (*I*^2^ = 34.4%, *p* = 0.123) - higher DII vs. lower DII, the pooled OR was 1.53 (95% CI: 1.39–1.69), with heterogeneity (*I*^2^ = 24.5%, *p* = 0.203) Subgroup and sensitivity analysis: - significant association between the highest DII vs. the lowest DII and case age; greater risk of UADT cancers among age > 60 yrs. (the pooled OR was 2.72, 95% CI: 2.17–3.41) than age ≤ 60 yrs. (the pooled OR was 1.81, 95% CI: 1.55–2.11, p*_regression_* = 0.017) - significant association between the highest DII vs. the lowest DII and study quality; greater risk of UADT cancers among studies with higher quality (the pooled OR was 2.82, 95% CI: 2.25–3.53) than lower quality (the pooled OR was 1.78, 95% CI: 1.52–2.08, p*_regression_* = 0.032) Dose-response association between DII and ESCC: - the pooled RR for a 1-unit increment in the DII was 1.18 (95% CI: 1.15–1.21) with heterogeneity (*I*^2^ = 82.1%, *p* = 0.126) The remaining results of the meta-regression analysis were not statistically significant.
		Tang et al./2018 [199]		
		Shivappa et al./2017 [200]		
		Lu et al./2016 [201]		
		Shivappa et al./2016 [202]		
		Shivappa et al./2016 [203]		
		Shivappa et al./2015 [204]		
		Shivappa et al./2015 [205]		
Hua et al. [53]/ 2020	2015–2018	Tang et al./2018 [199]	- 9 case-control studies (7202 cases/17,355 controls) - upper aerodigestive tract cancers (UADT) such as oesophageal (ESCC), oral (OSCC), nasopharyngeal (NPSCC), oropharyngeal (OPSCC), hypopharyngeal (HPSCC), laryngeal (LSCC) squamous cell cancers - 47–137-item FFQs (27–31 DII components) - DII/E-DII of the highest category vs. the lowest category - the covariates adjusted: cancer site and geographic region	The risk of UADT with categorical DII: - the highest DII vs. the lowest DII: the pooled OR was 2.27 (95% CI: 1.89–2.73), with heterogeneity (*I*^2^ = 60.2%, *p* < 0.001) The risk of ESCC with categorical DII: - the highest DII vs. the lowest DII: the pooled OR was 2.53 (95% CI: 1.74–3.68), with heterogeneity (*I*^2^ = 71.7%, *p* = 0.007) The risk of OSCC with categorical DII: - the highest DII vs. the lowest DII level: the pooled OR was 2.23 (95% CI: 1.73–2.86), with heterogeneity (*I*^2^ = 0.0%, *p* = 0.844) The risk of PSCC with categorical DII: - the highest DII vs. lowest DII level: the pooled OR was 2.02 (95% CI: 1.54–2.64), with heterogeneity (*I*^2^ = 20.3%, *p* = 0.275) The risk of LSCC with categorical DII: - the highest DII vs. the lowest DII level: the pooled OR was 2.05 (95% CI: 0.85–4.93), with heterogeneity (*I*^2^ = 85.6%, *p* = 0.001) Subgroup and sensitivity analysis: - significant association between the highest DII vs. the lowest DII and geographic region; the pooled OR was 2.11 (95% CI: 1.52–2.93) in Asia, the pooled OR was 2.19 (95% CI: 1.69–2.82) in Europe, the pooled OR was 3.01 (95% CI: 2.23–4.05) in USA
		Mazul et al./2018 [98]		
		Abe et al./2018 [55]		
		Shivappa et al./2017 [200]		
		Lu et al./2016 [201]		
		Shivappa et al./2016 [202]		
		Shivappa et al./2016 [203]		
		Shivappa et al./2015 [204]		
		Shivappa et al./2015 [205]		
Chen et al. [47]/ 2020	2015–2018	Tang et al./2018 [199]	- 8 case-control studies; 6 articles (1961 cases/3577 controls) - oesophageal (ESCC) squamous cell cancers - 47–137-item FFQs (23–36 DII components) - DII/E-DII of the highest category vs. the lowest category - subgroups analysis of the sources of the heterogeneity: published year, DII components, geographic region and study quality	The risk of ESCC with categorical DII: - the highest DII vs. the lowest DII: the pooled OR was 2.54 (95% CI: 1.90–3.40), with heterogeneity (*I*^2^ = 65.7%, *p* = 0.005) Subgroup and sensitivity analysis: - significant relationship between heterogeneity and published year; significant heterogeneity in research published in 2018 (*I*^2^ = 63.3%, *p* = 0.099) and no significant heterogeneity in studies published in 2015 (*I*^2^ = 35.7%, *p* = 0.183) - significant relationship between the heterogeneity and amount of DII components; significant heterogeneity in research with <30 food elements (*I*^2^ = 63.8%, *p* = 0.041); no significant heterogeneity in studies with ≥30 components (*I*^2^ = 25.1%, *p* = 0.261) - significant relationship between heterogeneity and geographic region; significant heterogeneity in research from Asia (*I*^2^ = 73.0%, *p* = 0.024) and no significant heterogeneity in studies from Europe (*I*^2^ = 25.1%, *p* = 0.261)
		Abe et al./2018 [55]		
		Shivappa et al./2017 [200]		
		Lu et al./2016 [201]		
		Shivappa et al./2015 [204]		
		Shivappa et al./2015 [205]		
Jayedi et al. [207]/2018	2015–2016	Lu et al./2016 [201]	- 5 case-control studies (1167 cases/3332 controls) - oesophageal (ESCC), laryngeal (LSCC), nasopharyngeal (NPSCC) squamous cell cancers - 120–125-item FFQs (27–31 DII components) - DII (1) and a 1-unit increment in the DII (2)	- DII: ESCC, LSCC, NPSCC—significant positive associations (results NR; OR/HR/RR value not given) - the pooled RR for a 1-unit increment in the DII was 1.24 (95% CI: 1.10–1.38) for ESCC - the pooled RR for a 1-unit increment in the DII was 1.27 (95% CI: 1.15–1.40) for LSCC - the pooled RR for a 1-unit increment in the DII was 1.09 (95% CI: 1.02–1.16) for NPSCC
		Shivappa et al./2016 [202]		
		Shivappa et al./2016 [203]		
		Shivappa et al./2015 [204]		
		Shivappa et al./2015 [205]		

DII: Dietary inflammatory index; E-DII, energy adjusted DII; NR: not reported; OR/HR/RR: odds ratio/hazard ratio/risk ratio; CI: confidence interval; FFQs, food frequency questionnaires; SES: socio-economic status; BMI: body mass index; NOS: the Newcastle-Ottawa scale; OSCC: oral squamous cell cancer; PSCC: pharyngeal squamous cell cancer; OPSCC: oropharyngeal squamous cell cancer; LSCC: laryngeal squamous cell carcinoma; NPSCC: nasopharyngeal squamous cell carcinoma; UADT: upper aerodigestive tract squamous cell carcinoma.

## Data Availability

No new data were created or analyzed in this study. Data Sharing is not applicable to this article.
